# Alternative splicing of *BCL-x* is controlled by RBM25 binding to a G-quadruplex in *BCL-x* pre-mRNA

**DOI:** 10.1093/nar/gkad772

**Published:** 2023-10-09

**Authors:** Ronan Le Sénéchal, Marc Keruzoré, Alicia Quillévéré, Nadège Loaëc, Van-Trang Dinh, Oksana Reznichenko, Pedro Guixens-Gallardo, Laurent Corcos, Marie-Paule Teulade-Fichou, Anton Granzhan, Marc Blondel

**Affiliations:** Univ Brest; Inserm UMR1078; Etablissement Français du Sang (EFS) Bretagne; CHRU Brest, Hôpital Morvan, Laboratoire de Génétique Moléculaire, 22 avenue Camille Desmoulins, F-29200 Brest, France; Univ Brest; Inserm UMR1078; Etablissement Français du Sang (EFS) Bretagne; CHRU Brest, Hôpital Morvan, Laboratoire de Génétique Moléculaire, 22 avenue Camille Desmoulins, F-29200 Brest, France; Univ Brest; Inserm UMR1078; Etablissement Français du Sang (EFS) Bretagne; CHRU Brest, Hôpital Morvan, Laboratoire de Génétique Moléculaire, 22 avenue Camille Desmoulins, F-29200 Brest, France; Univ Brest; Inserm UMR1078; Etablissement Français du Sang (EFS) Bretagne; CHRU Brest, Hôpital Morvan, Laboratoire de Génétique Moléculaire, 22 avenue Camille Desmoulins, F-29200 Brest, France; Univ Brest; Inserm UMR1078; Etablissement Français du Sang (EFS) Bretagne; CHRU Brest, Hôpital Morvan, Laboratoire de Génétique Moléculaire, 22 avenue Camille Desmoulins, F-29200 Brest, France; Chemistry and Modelling for the Biology of Cancer (CMBC), CNRS UMR9187, Inserm U1196, Institut Curie, Université Paris Saclay, F-91405 Orsay, France; Chemistry and Modelling for the Biology of Cancer (CMBC), CNRS UMR9187, Inserm U1196, Institut Curie, Université Paris Saclay, F-91405 Orsay, France; Univ Brest; Inserm UMR1078; Etablissement Français du Sang (EFS) Bretagne; CHRU Brest, Hôpital Morvan, Laboratoire de Génétique Moléculaire, 22 avenue Camille Desmoulins, F-29200 Brest, France; Chemistry and Modelling for the Biology of Cancer (CMBC), CNRS UMR9187, Inserm U1196, Institut Curie, Université Paris Saclay, F-91405 Orsay, France; Chemistry and Modelling for the Biology of Cancer (CMBC), CNRS UMR9187, Inserm U1196, Institut Curie, Université Paris Saclay, F-91405 Orsay, France; Univ Brest; Inserm UMR1078; Etablissement Français du Sang (EFS) Bretagne; CHRU Brest, Hôpital Morvan, Laboratoire de Génétique Moléculaire, 22 avenue Camille Desmoulins, F-29200 Brest, France

## Abstract

*BCL-x* is a master regulator of apoptosis whose pre-mRNA is alternatively spliced into either a long (canonical) anti-apoptotic Bcl-xL isoform, or a short (alternative) pro-apoptotic Bcl-xS isoform. The balance between these two antagonistic isoforms is tightly regulated and overexpression of Bcl-xL has been linked to resistance to chemotherapy in several cancers, whereas overexpression of Bcl-xS is associated to some forms of diabetes and cardiac disorders. The splicing factor RBM25 controls alternative splicing of *BCL-x*: its overexpression favours the production of Bcl-xS, whereas its downregulation has the opposite effect. Here we show that RBM25 directly and specifically binds to GQ-2, an RNA G-quadruplex (rG4) of *BCL-x* pre-mRNA that forms at the vicinity of the alternative 5′ splice site leading to the alternative Bcl-xS isoform. This RBM25/rG4 interaction is crucial for the production of Bcl-xS and depends on the RE (arginine-glutamate-rich) motif of RBM25, thus defining a new type of rG4-interacting domain. PhenDC3, a benchmark G4 ligand, enhances the binding of RBM25 to the GQ-2 rG4 of *BCL-x* pre-mRNA, thereby promoting the alternative pro-apoptotic Bcl-xS isoform and triggering apoptosis. Furthermore, the screening of a combinatorial library of 90 putative G4 ligands led to the identification of two original compounds, PhenDH8 and PhenDH9, superior to PhenDC3 in promoting the Bcl-xS isoform and apoptosis. Thus, favouring the interaction between RBM25 and the GQ-2 rG4 of *BCL-x* pre-mRNA represents a relevant intervention point to re-sensitize cancer cells to chemotherapy.

## Graphical Abstract

**Graphical Abstract:**
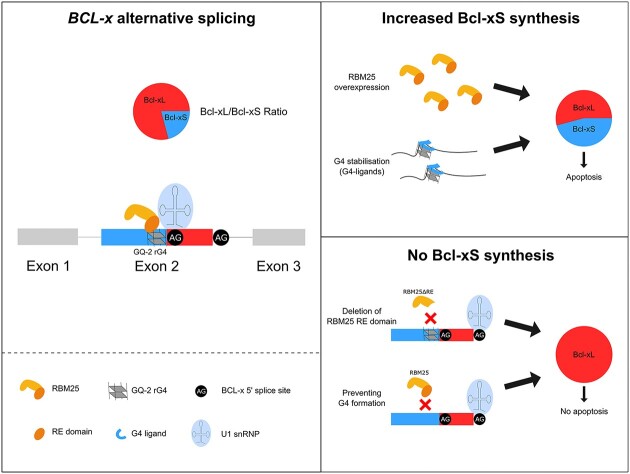

## Introduction

Alternative splicing (AS), discovered simultaneously with splicing >40 years ago (1–3), is prevalent in multicellular eukaryotes in which it is the main mechanism allowing one gene to produce various proteins, thereby increasing the complexity of the proteome from a limited number of genes. In addition, AS plays key roles in determining tissue- and species-specific differentiation patterns. AS is regulated by multiple co- and post-transcriptional regulatory mechanisms and can play a causal role in various hereditary disorders as well as cancers (4,5).

The *BCL-x* gene (aka *BCL2-like 1*, *BCL2L1*) encodes Bcl-x (6), a member of the Bcl-2 (B-cell lymphoma) family of proteins that are major regulators of apoptosis, a highly conserved and essential programmed cell death process that eliminates unwanted cells, including damaged cells, lifelong including during development (7). Bcl-x is a mitochondrial transmembrane protein that regulates the intrinsic apoptosis pathway. Owing to the existence of two alternative 5′ splice sites (5′ss) in its exon 2, the *BCL-x* pre-mRNA is alternatively spliced into either a long (canonical) anti-apoptotic Bcl-xL isoform, or a short (alternative) pro-apoptotic Bcl-xS isoform (6). The ability of Bcl-xL to inhibit apoptosis involves several mechanisms that include inhibition of Bax-induced mitochondrial outer membrane permeabilization (MOMP), a key step in apoptosis execution (8). Besides this canonical function, Bcl-xL has been also shown to remotely control apoptosis by inhibiting IP3R channels and therefore the release of Ca^2+^ from the endoplasmic reticulum (RE) (9,10), the transfer of Ca^2+^ from the RE to the mitochondria being also involved in MOMP (11). Bcl-xS has been proposed to activate apoptosis by inhibiting Bcl-xL via a direct interaction (8). Hence, depending on the balance between Bcl-xL and Bcl-xS, *BCL-x* can either be pro- or anti-apoptotic. As such, the balance between these two antagonistic isoforms is tightly regulated and overexpression of the anti-apoptotic Bcl-xL isoform has been linked to resistance to chemotherapy in several cancers (12,13), whereas overexpression of the pro-apoptotic Bcl-xS isoform is associated to some forms of diabetes and cardiac disorders (14,15). Therefore, pharmacological means to switch the splicing in favour of Bcl-xS may have therapeutic benefits for a number of cancers, in particular those resistant to chemotherapy due to Bcl-xL overexpression. Conversely, favouring Bcl-xL represents a possible intervention point for diseases linked to Bcl-xS overexpression such as some forms of diabetes. In line, splice switching oligonucleotides (SSO) that block the 5′ss leading to the long canonical Bcl-xL isoform have been developed and shown to reduce the overall cell viability of a variety of cancer cell lines (16–19). In addition, inhibitors of SR kinases that phosphorylate serine-arginine-rich (SR) proteins, have been tested (20,21) with the inherent limitation that alterations in the phosphorylation of SR proteins, which play prominent roles in pre-mRNA constitutive and alternative splicing decisions, are likely to have widespread effects on the splicing of many genes.

G-quadruplex (G4) are non-canonical secondary structures that may assemble in guanine-rich (G-rich) DNA or RNA. G4 are formed by the stacking of at least two G-quartets, each representing a planar arrangement of four guanines connected by Hoogsteen hydrogen bonds and stabilized by a central cation, most often K^+^. G4 structures within G-rich DNA or RNA have been implicated in gene regulation where they can affect transcription (22), splicing (23–25) or translation (26–32).

G4 have been shown to be involved in *BCL-x* alternative splicing (33,34). Indeed, the *BCL-x* pre-mRNA contains a number of G-rich sequences that can potentially form at least six RNA G4 (rG4) as predicted *in silico*. In addition, thanks to FOLDeR, a new strategy based on footprinting of long 7-deazaguanine-substituted RNA, the formation of two of these rG4 was confirmed *in vitro* under functional conditions: one (GQ-2) lying in close proximity with the 5′ss leading to the short (alternative) pro-apoptotic Bcl-xS isoform (xS 5′ss) while the other (GQ-5) is overlapping the 5′ss leading to the long (canonical) anti-apoptotic Bcl-xL isoform (xL 5′ss) (33). In addition, the ability of GQ-2 to adopt a G4 conformation has been recently confirmed by various biophysical experiments (34,35).

Multiple splice factors and signalling pathways have been implicated in the regulation of *BCL-x* pre-mRNA (alternative) splicing. Most of these proteins are general splice factors and include SRSF1 (36,37), SRSF2 (38), SRSF 3 and 7 (39), SRSF9 (36) and SRSF10 (40). The following hnRNP have also been involved: A1 (37), K (41), F/H (42,43) and PTBP1 (39) as well as other RNA-binding protein: Sam68 (37), SF3B1 (44), TRA2 (39), RBM4 (45), RBM10 (46), RBM11 (47) and RBM25 (48). RBM25 (also known as RED120) belongs to a family of RNA-binding proteins whose members share an arginine-glutamate-rich (RE) central region and a C-terminal proline-tryptophan-isoleucine (PWI) motif. RBM25 also contains an N-terminal RNA recognition motif (RRM). RBM25 associates with various splicing factors that include SRm160/300 and U snRNAs, as well as with spliced RNA (49). Overexpression of RBM25 has been shown to increase the expression of the alternative pro-apoptotic Bcl-xS isoform and to trigger apoptosis whereas its downregulation leads to accumulation of the canonical anti-apoptotic Bcl-xL isoform (48).

Here we show that RBM25 directly and specifically binds in a G4 structure-dependent manner to the GQ-2 rG4 of the *BCL-x* pre-mRNA, which forms in the vicinity of the alternative 5′ splice site that leads to the alternative Bcl-xS isoform (xS 5′ss). We also show that this RBM25/rG4 interaction is crucial for the production of Bcl-xS and depends on the central RE motif of RBM25, thus defining a new type of rG4-interacting domain. This RBM25/rG4 interaction is druggable as PhenDC3, a benchmark G4 ligand, stabilizes GQ-2 rG4 *in vitro* and enhances the binding of RBM25 to the GQ-2 rG4 of the *BCL-x* pre-mRNA, hence promoting the alternative pro-apoptotic Bcl-xS isoform and apoptosis. Therefore, favouring the interaction between RBM25 and the GQ-2 rG4 of *BCL-x* pre-mRNA represents a relevant intervention point to re-sensitize cancer cells to chemotherapy, whereas interfering with this interaction may represent a therapeutic avenue for diseases associated with Bcl-xS overexpression, such as some forms of diabetes. For this reason, we have also screened a library of 90 putative G4 ligands related to PhenDC3 (50) and identified PhenDH8 and PhenDH9, two original G4-ligands, that are more potent than PhenDC3 to promote the alternative pro-apoptotic Bcl-xS isoform and apoptosis.

## Material and methods

### *In vitro* characterization of RNA oligonucleotides

RNA oligonucleotides (Eurogentec, HPLC-purified) were dissolved in deionized water at a concentration of 100 μM and further diluted to a concentration of 4 μM with a lithium cacodylate buffer (10 mM, pH 7.2) supplemented with 100 mM KCl, 100 mM LiCl or their mixture to obtain the desired concentration of K^+^, in a sample volume of 1 mL. Thermal denaturation spectra (TDS) were recorded as described by Mergny *et al.* (51) using sample temperatures of 20 and 80°C. Ultraviolet (UV) melting profiles were recorded using a heating ramp of 0.2°C min^−1^ and a detection wavelength of 295 nm on a Cary 300 Bio spectrophotometer (Agilent) equipped with a Peltier-controlled multicell holder, and *T*_m_ values were obtained by the first-derivative analysis of melting transitions. FRET melting experiments were performed according to the published procedure (52), using 5′-FAM / 3′-TAMRA labelled GQ2 oligonucleotide (F-GQ2-T, Eurogentec).

### Plasmid constructions

All the RBM25 plasmids were generated using standard procedures. The T4 DNA ligase was obtained from Promega and the restriction enzymes were purchased from New England Biolabs. RBM25 was amplified from complementary DNA (cDNA) prepared from extracts of H1299 cells with primers 5′-GCGCGCGGTACCATGTACCCATACGATGTTCCAGATTACGCTTCTTTTCCACCTCATTTGAATCGC-3′ and 5′-GCGCGCGCGGCCGCTTACTTCACAAGACCAATTTTCTTGGC-3′. The PCR fragment was cloned into KpnI and NotI cloning sites of pcDNA 3.1(+) vector. The generated construction was verified by PCR amplification, restriction enzymes digestion and sequencing.

The Bcl-x 672 minigenes synthesis was carried out by GenScript. On reception, the minigenes were amplified in bacteria. Plasmid extraction was performed using the NucleoBond^®^ Xtra Midi EF kit protocol (Macherey-Nagel). The plasmids were verified by PCR amplification and sequencing.

### Cell culture, treatment and transfection

H1299 cells were derived from metastatic lymph node from lung carcinoma. HeLa cells were derived from a cervical carcinoma. A549 cells were derived from a lung carcinoma. H1299, HeLa and A549 cells were purchased from ATCC and cultured in RPMI-1640 supplemented with 10% foetal bovine serum and 2 mM l-glutamine. Transfections were performed using GeneJuice^®^ (Merck) or FuGENE^®^ HD (Promega) transfection reagent according to the manufacturers’ protocols. Where indicated, cells were treated with various concentrations of G4 ligands, doxorubicin (Sigma) or with DMSO (compounds vehicle) for 24 or 48 hours.

### Protein extraction from mammalian cells

Whole cells at 75–90% confluency were harvested, washed with 1x phosphate buffered saline (PBS) and suspended into lysis buffer (20 mM HEPES pH 7.5, 50 mM β-glycerolphosphate, 1 mM EDTA pH 8, 0.5 mM Na_3_VO_4_, 100 mM KCl, 10% glycerol, 1% Triton and anti-proteases cocktail (Roche 11697498001)). These cell suspensions were mechanically lysed before centrifugation at 16 000*g* for 20 min at 4°C and protein concentration was determined using a Bradford assay.

### Western blotting

Equal protein quantities and volumes of all samples were loaded and run on Bolt or NuPAGE^tm^ 10% Bis-Tris Protein gels (Invitrogen), then transferred onto 0.45 μM nitrocellulose membrane (GE Healthcare). Membranes were blocked in 1x PBS, 0.1% Igepal and 3% bovine serum albumin (BSA), and incubated with the indicated primary antibodies: mouse anti-GAPDH (Abcam ab125247, 1/5000), rat anti-HA (Roche 11867423001, 1/2000), rabbit anti-RBM25 (Merck HPA003025, 1/1000) or rabbit anti-Bcl-xL (CellSignaling no. 2764, 1/500). The membranes were then washed with fresh 1x PBS 0.1% Igepal and incubated with the indicated secondary antibodies conjugated to horseradish peroxidase: rabbit anti-mouse (Dako P0161, 1/3000), swine anti-rabbit (Dako P0217, 1/2000) or goat anti-rat (Millipore AP136P, 1/3000). The membranes were washed afresh and analysed by enhanced chemiluminescence in the following buffer (Tris-base pH 8.5, 12.5 nM coumaric acid, 2.25 nM luminol and 0.15% H_2_O_2_) using a Vilber-Lourmat Photodocumentation Chemistart 5000 imager. Relative proteins levels for each sample were normalized to GAPDH protein levels using the ImageJ software.

### RNA and RNA pulldowns

RNA pulldowns experiments were performed as previously described (27,31). Briefly, the following G-quadruplex forming RNA oligonucleotides 3′-tagged with tetraethylene glycol (TEG)-Biotin were used:

- GQ-1 GGGAGGCAGGCGACGAGUUUGAACUGCGGUACCGGCGGGCA-3′-TEG-Biotin,
- GQ-2 GGGAUGGGGUAAACUGGGGUCGCAUUGUGG-3′-TEG-Biotin,
- GM-2 GAGAUGAGGUAAACUGAGGUCGCAUUGUGG-3′-TEG-Biotin,
- GQ-5 GGAUCCAGGAGAACGGCGGCUGG-3′-TEG-Biotin and
- GM-5 GGAUCCAGAAGAACGGCGGCUGA-3′-TEG-Biotin.

The G-quadruplexes were formed by heating the RNA oligonucleotides at 95°C for 5 min then cooling down to 4°C at a rate of 2°C per minute in folding buffer (10 mM Tris-HCl pH 7.5, 0.1 mM EDTA) in the presence of 100 mM KCl, or in the presence of 100 mM LiCl. To avoid unspecific binding, high affinity streptavidin Sepharose beads (GE Healthcare, 28985799) were incubated in 1 mL of blocking buffer (10 mM Tris-HCl, pH 7.5, 100 mM KCl, 0.1 mM EDTA, 1 mM DTT, 0.01% Triton, 0.1% BSA, 0.02% *S. cerevisiae* tRNAs (Sigma-Aldrich 10109495001)) for 1 h at 4°C on a rotating wheel. An amount of 10 μg of each batch of folded biotinylated RNA oligonucleotides was incubated with 50 μL of solution containing the streptavidin sepharose beads for 90 min at 4°C on a rotating wheel. Next, 500 μg of cell extracts or 200 ng of recombinant RBM25 were treated with 200 U/mL of RNase Inhibitor (NEB, M0307S) for 90 min at 4°C on a rotating wheel. These extracts were then incubated with the RNA oligonucleotides bound to the streptavidin beads for 90 min at room temperature. Beads were then washed five times with lysis buffer and then with lysis buffer with increasing KCl or LiCl concentrations (200 to 800 mM). Protein still bound to beads after the washes were eluted using 2x loading buffer (2x Laemmli Buffer with 5% β-mercaptoethanol) and analysed by western blotting with antibodies raised against RBM25 or HA.

The same protocol was applied for DNA pulldowns, except that G-quadruplex forming DNA oligonucleotides 3′-tagged with TEG-Biotin were used.

### Proximity ligation assay adapted to protein–RNA interactions

An adaptation of proximity ligation assay (PLA) to detect protein–RNA interactions (31,53) has been used. Briefly, cells were fixed in 1x PBS, 4% paraformaldehyde for 20 min and permeabilized for 10 min with 0.4% Triton X-100, 0.05% CHAPS. The Bcl-x-mRNA-digoxigenin probe (5′-CCATTGTCCAAAACACCTGCTCACTCACTGAGTCTCGTCTCTGGAAAAA-3′) at a quantity of 100 ng per well was denatured 5 min at 80°C. The probe hybridization reaction was carried out in 40 μL of hybridization buffer (10% formamide, 2X SSC, 0.2 mg/ml *E. coli* tRNA, 0.2 mg/ml salmon sperm DNA and 2 mg/ml BSA). Fixed cells were washed and blocked in the blocking solution (1x PBS, 3% BSA, 0.1% Saponine) before incubation with the primary antibodies (anti-digoxigenin, Sigma 1/200 and anti-RBM25, Merck 1/4000). The PLA reaction was performed according to the manufacturer's protocol using the Duolink^®^ PLA *in situ* kit, PLA probe anti-rabbit Plus, PLA probe anti-mouse Minus and the FarRed *in situ* detection reagent, all purchased from Sigma. The results were analysed using a Zeiss Axio Imager M2 and the ImageJ software. All PLA experiments were performed at least twice independently and the following controls were implemented: with sense mRNA probe or without primary antibodies. The PLA method with the use of Flag Bcl-x minigene was carried out as follow: H1299 cells were plated and transfected with 400 ng of Flag Bcl-x minigene plasmid 24 h before the paraformaldehyde fixation process described before was performed. The Flag-Bcl-x-mRNA-digoxigenin probe (5′-CCATGGGGATCACCTCCCGCTTGTCGTCATCGTCTTTGTAGTAAAAA-3′) at a quantity of 100 ng per well was denatured 5 min at 80°C prior to the hybridization step. After the hybridization of the probe, an incubation with the primary antibodies (anti-digoxigenin Sigma 1/200 and anti-RBM25 Merck 1/1000) was performed. The remaining steps of the protocol are as described before. The PLA to assess the interaction between RBM25 and G4s used the same protocol (without the hybridization step) except that the anti-G4 (BG4) antibody (Absolute Antibody, 1/200) was used together with the anti-RBM25 antibody (Merck 1/4000).

### RNA extraction and semi-quantitative RT–PCR

Total A549 or HeLa cellular RNA were extracted using the RNeasy Plus kit (Qiagen). cDNA synthesis was carried out using 500 ng of RNA and the SuperScript™ IV Reverse Transcriptase (Invitrogen) together with oligo(dT)_20_ primer (Invitrogen). cDNA samples were analysed using semi-quantitative PCR using the MasterMix OneTaq Polymerase (NEB). Bands were quantified using the ImageJ software to determine Bcl-xS percentage.

For Bcl-x 672 minigene splicing analysis, PCR was performed using P1 primer specific to pcDNA 3.1 (+) plasmid (5′-TAGAAGGCACAGTCGAGG-3′) and P2 primer specific to *BCL-x* (5′-GGGAGGTGATCCCCATGGCAG-3′).

For endogenous *BCL-x* splicing analysis, PCR was performed using P2 primer (5′-GGGAGGTGATCCCCATGGCAG-3′) and P3 primer specific to *BCL-x* (5′-GGCCACAGTCATGCCCGTCAG-3′).

### Immunofluorescence

H1299 cells were plated on 13 mm-diameter coverslips in 24-well plates and transfected with HA-RBM25-FL or HA-RBM25ΔRE plasmids. At 24 h post-transfection, cells were fixed with 4% paraformaldehyde in PBS for 20 min and permeabilized with PBS 0.4%, Triton X-100, CHAPS 0.05% for 10 min at room temperature. After incubation in blocking buffer (1x PBS, 3% BSA, 0.1% Saponin) for 30 min at room temperature, samples were incubated with a mouse polyclonal anti-HA antibody (a kind gift from Borek Vojtesek, Masaryk Memorial Cancer Institute, Brno, Czech Republic) at 1/1000 and a rabbit anti-RBM25 antibody (Merck HPA003025) at 1/500 for 2 h at room temperature, followed by 1 h incubation with 1/500 dilutions of the goat anti-mouse immunoglobulin G (IgG) secondary antibody conjugated to Alexa Fluor^®^ 594 and of the goat anti-rabbit immunoglobulin G (IgG) secondary antibody conjugated to Alexa Fluor^®^ 488 (both purchased from Invitrogen). Both antibodies were diluted in blocking buffer. 4',6-Diamidino-2-phenylindole (DAPI) was used for nuclear counterstaining and the images were taken using a Zeiss Axio Imager M2.

### Caspase activity assay

About 4000 A549 cells were plated in 0.1 mL of medium per well in 96-well flat-bottom plates. After 18 h, cells were exposed to the indicated compounds at the indicated concentrations or to dimethylsulphoxide (DMSO; vehicle) as a negative control. After 48 h of treatment, the Caspase 3/7 activity was determined using the Caspase Glo^®^ 3/7 Assay (Promega) according to the manufacturer's protocol.

### MTT assay

About 4000 A549 cells were plated in 0.1 ml of medium per well in 96-well flat-bottom plates. After 18 h, cells were exposed to the indicated compounds at the indicated concentrations or to DMSO (vehicle) as a negative control. After 48 h of treatment, 10 μL of MTT solution (5 mg/mL in PBS (pH 7.4), CT01-5, Merck Millipore) was added to each well and cells were incubated for 4 h. Next, 100 μL of an isopropanol solution containing 0.1 N HCl and 10% (v/v) Triton X100 was added to each well to dissolve the formazan crystals, and the absorbance at 570 nm was then measured.

## Results

### RBM25 selectively binds to GQ-2 rG4 in a G4 structure-dependent manner

**Figure 1.**
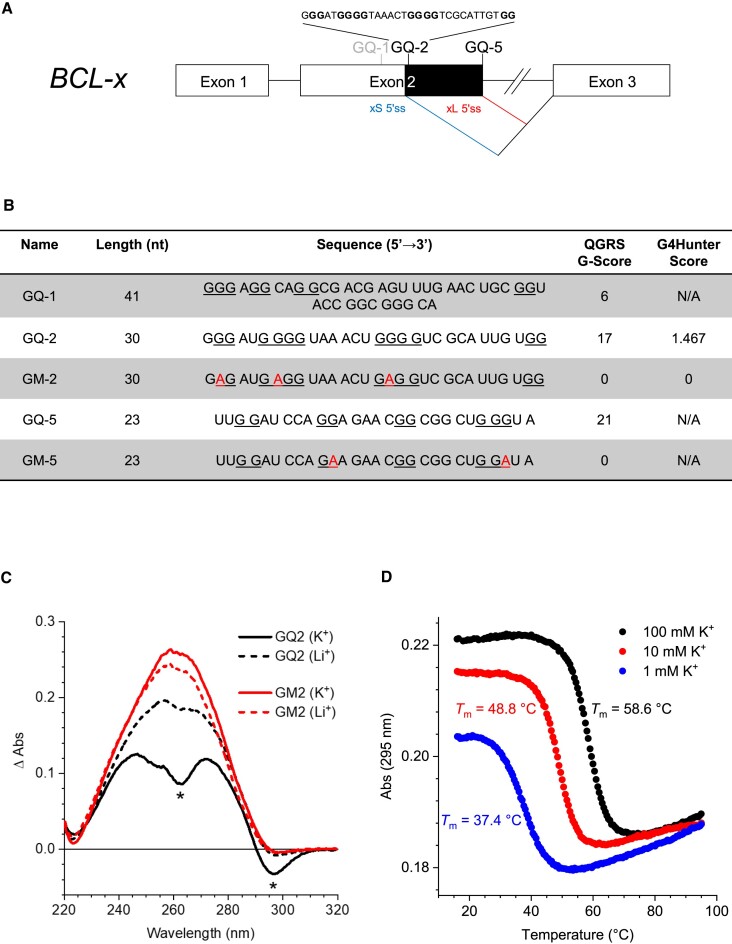
GQ-2 is an rG4 that may form in close proximity to the alternative splice site that leads to the synthesis of Bcl-xS, the short alternative pro-apoptotic isoform of Bcl-x. (**A**) Schematic representation of the *BCL-x* gene. The two alternative 5′ splice sites in the exon 2 are indicated (xS 5′ss is highlighted in blue and xL 5′ss in red) as well as the two putative rG4 (GQ-2 and GQ-5) that may assemble at close proximity to these two splice sites. GQ-1 (written in grey) is a putative quadruplex sequence (PQS), as predicted by G4-finder, which has not been confirmed *in vitro*. (**B**) Sequences of the various RNA oligonucleotides that have been used for RNA pulldown experiments. The various sequences, together with their predicted propensity to form G4 (as determined using two different software: G-score and G4-hunter) are indicated and the guanines potentially important for G4 formation are underlined. (**C**) TDS (ΔAbs = Abs (80°C) − Abs (20°C)) of GQ-2 and GM-2 RNA oligonucleotides (4 μM) in 10 mM lithium cacodylate buffers supplemented with 100 mM KCl (solid lines) or 100 mM LiCl (dashed lines). G4-characteristic peaks are indicated with asterisks. (**D**) UV-melting analysis of GQ-2 (4 μM) in the presence of various concentrations of K^+^ as indicated.

Whereas exons are enriched in guanines in eukaryotes (54), the presence of G4 seems to have been counter selected (55,56). However, as stated before, the formation of two rG4 from two potential quadruplex-forming sequences (PQS) in the exon 2 of the *BCL-x* gene have been confirmed *in vitro* under functional conditions (33). One of these two rG4, GQ-2, is localized at the close vicinity of the 5′ss leading to the short alternative pro-apoptotic Bcl-xS isoform (xS 5′ss) whereas the other, GQ-5, is overlapping the 5′ss leading to the long canonical anti-apoptotic Bcl-xL isoform (xL 5′ss) (Figure 1A). The sequence of these two PQS is shown in Figure 1B, together with the sequence of GQ-1, a PQS upstream of GQ-2 whose formation was not confirmed *in vitro* under physiological conditions, and of GM-2 and GM-5, two mutated versions of GQ-2 and GQ-5, respectively, in which a minimal number of guanines (3 and 2, respectively) involved in G4 formation were replaced by adenines to prevent G4 formation, as predicted using the G4Killer software that has been developed to design mutations crippling G4 propensity (57). The capacity of GQ-2 fragment to form rG4 structure *in vitro* has recently been demonstrated by Bhogadia *et al.* through CD, ^1^H nuclear magnetic resonance and gel electrophoresis experiments (35). Here, we additionally assessed the capacity of GQ-2 and its variants to form G4 structures *in vitro* using TDS and UV melting experiments. In agreement with previously published results and bioinformatics analysis (QGRS Mapper (58) and G4Hunter (59), cf. the corresponding scores in Figure 1B), we found that, in the presence of K^+^, but not Li^+^, GQ-2 adopted a G4 conformation evidenced by characteristic negative TDS peaks at 295 and 260 nm, whereas GM-2 was unable to do so, even in the presence of K^+^ (Figure 1C). The UV-melting profile of GQ-2 featured a negative transition at 295 nm, with melting temperatures being strongly dependent on K^+^ concentration (*T*_m_ = 37.4, 48.8 and 58.6°C in the presence of 1, 10 or 100 mM KCl, respectively, Figure 1D), which is a strong hallmark of G4 formation (60). To get preliminary insight into the rG4 structure formed by GQ-2, we studied three additional sequence variants in which isolated guanines G22 or/and G27 were replaced by adenines (GQ2-A22, GQ2-A27 and GQ2-A22A27, respectively). Their thermal difference spectra (Supplementary Figure 1) revealed G4-characteristic negative peaks at 295 and 260 nm in the presence of K^+^, but not Li^+^, giving evidence that these variants were also able to form rG4 structures. In addition, UV-melting analysis (Supplementary Figure 2) indicated that thermal stability of these variants was only marginally affected compared to GQ-2, indicating that guanines G22 and G27 do not participate in formation of the rG4 core. However, in the case of GQ2-A27 and, especially, GQ2-A22A27, the amplitude of G4-specific melting transition was reduced at low K^+^ concentration (1 mM), suggesting possible formation of alternative structures in addition to rG4. Altogether, these observations suggest that GQ2 forms a two-layer rG4 structure without bulge.

**Figure 2.**
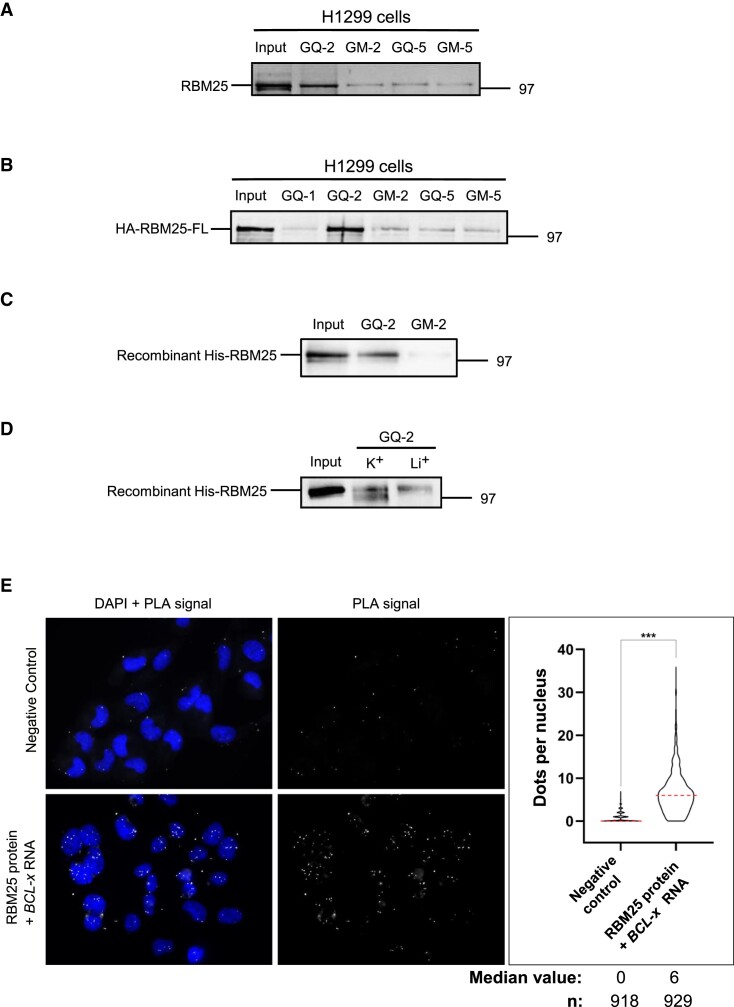
RBM25 selectively binds to GQ-2 rG4 in a G4-dependent manner (**A**) RNA pulldown of extracts from H1299 cells using the indicated RNA oligonucleotide matrices. The proteins still bound after an 800 mM KCl wash were eluted and analysed by sodium dodecyl sulphate-polyacrylamide gel electrophoresis (SDS–PAGE) and western blot using an anti-RBM25 antibody to reveal endogenous RBM25. Gel represents *n*≥ 3. (**B**) Same RNA pulldown experiment as in (**A**) except that extracts from H1299 cells transfected by a plasmid allowing expression of HA-tagged RBM25 (HA-RBM25-FL) were used. Exogenous HA-RBM25-FL was revealed using an anti-HA antibody. Gel represents *n*≥ 3. (**C**) The same RNA pulldown experiment as in (**A**) and (**B**) except that a recombinant polyhistidine-tagged RBM25 protein (His-RBM25) was used instead of extracts from H1299 cells. Gel represents *n*≥ 3. (**D**) Control experiment using the GQ-2 matrix folded in the presence of 100 mM KCl (that favours G4 formation) or 100 mM LiCl (that does not favour formation of G4), as indicated, and recombinant His-RBM25. Gel represents *n*≥ 3. (**E**) Adaptation of the PLA to monitor the RBM25 protein-*BCL-x* mRNA interaction performed in H1299 cells natively expressing RBM25 protein and *BCL-x* mRNA. Microscopy images of H1299 cells analysed using a probe specifically hybridizing to the *BCL-x* RNA (lower panels) or not (upper panels, negative controls) as indicated. Nuclei were revealed by DAPI staining and appear in blue. White dots (PLA signals) indicated interaction (close proximity) between RBM25 protein and *BCL-x* RNA. The graph on the right indicates the number of PLA dots per cell in each condition. Data from three biological replicates, at least 250 cells per sample, were analysed by a non-parametric Mann–Whitney's test using the GraphPad Prism 8 Software (***P< 0.0001).

Next, to identify protein(s) presenting potential affinity for GQ-2 and/or GQ-5 rG4, we performed RNA pulldown assays as previously described (31,61). Briefly, this assay is based on the use of an RNA oligonucleotide containing a sequence that can form G4, 3′-conjugated to biotin through a TEG spacer, which can therefore be precipitated by magnetic sepharose beads conjugated to streptavidin. This way, proteins with affinity for the selected RNA-G4 can be precipitated and detected by western blotting. Interestingly, RBM25 has recently been identified in a comprehensive approach as one of 281 potential G4-related proteins in MM231 cells, thanks to an original method based on a photoactive G4 ligand (62). Hence, in addition to regulate *BCL-x* alternative splicing as presented in the introduction, RBM25 is a potential G4-binding protein. For these reasons we first determined, using RNA pulldown assays, if endogenously expressed RBM25 binds, or not, to one or the other of the following matrices: GQ-2, GM-2, GQ-5 and GM-5 RNA oligonucleotides conjugated to biotin. For this purpose, we used total protein extracts prepared from H1299 cells. As shown in Figure 2A, an efficient binding of endogenous RBM25 was observed only on the GQ-2 matrix. This binding mostly depends on G4 since only a residual binding was observed with the GM-2 sequence unable to adopt a G4 structure, similar to the residual binding observed with GQ-5 and GM-5 matrices.

We then repeated these RNA pulldown experiments using total protein extracts prepared from H1299 cells transfected with a plasmid allowing expression of HA-tagged RBM25 (HA-RBM25-FL) (Figure 2B). As an additional control, we used biotin-conjugated GQ-1 RNA oligonucleotide. We found that exogenously expressed HA-RBM25 efficiently binds to GQ-2 whereas only a residual binding was observed on all the other matrices.

The binding of RBM25 to the GQ-2 rG4 may be indirect and involve other protein(s). To determine whether the specific binding of RBM25 is direct or indirect, we repeated the same RNA pulldown experiments using purified recombinant His-tagged RBM25 produced in bacteria (His-RBM25). We obtained the same result as His-RBM25 efficiently binds to GQ-2, whereas only a residual binding was observed on GM-2 control matrix that does not form G4 (Figure 2C). This result shows that RBM25 directly binds to GQ-2 rG4. We also performed DNA pulldown experiments to determine whether RBM25 is able to bind to DNA G-quadruplexes (dG4). For this purpose, we have used the same protocol than the one for RNA pulldown, except that the following DNA matrices were employed: GQ-2, GM-2, BCL-2, BCL-2mut, MYC and MYCmut DNA oligonucleotides 3′-conjugated to biotin that contain, or not for the mutants, a sequence that can form dG4. As shown in Supplementary Figure 3, RBM25 is able, at least *in vitro*, to bind to these various dG4, compared to their mutated versions that cannot form dG4. Of note, the binding of RBM25 appears less efficient on GQ-2 dG4 than on GQ-2 rG4 suggesting that RBM25 may have more affinity for rG4 than for dG4.

To confirm that the binding on the GQ-2 matrix was G4-dependent, we performed an additional control experiment solely based on the GQ-2 matrix folded in the presence of K^+^ (100 mM KCl), which allows G4 formation, or Li^+^ (100 mM LiCl), which does not allow efficient G4 formation. As shown in Figure 2D, the binding of recombinant His-RBM25 is more efficient in the condition where G4 formation is favoured (K^+^), thereby confirming that the interaction of RBM25 with the GQ-2 rG4 is indeed G4 structure-dependent.

Finally, we performed PLA to assess whether, and where in the cell, endogenously expressed RBM25 interacts with endogenously expressed *BCL-x* mRNA. For this purpose, we used a recently described adaptation of PLA to monitor protein–RNA interactions (53). As shown in Figure 2E, we observed a clear nuclear signal (median value of six dots per cell, most of them being nuclear, as compared to zero dots per cell as a median value for the negative control). This confirms *in cellulo* the interaction between RBM25 protein and *BCL-x* RNA (both partners being expressed at their physiological level) and indicates that this interaction takes place in the nucleus, as expected for interactions between splice proteins and pre-mRNA. In addition, to assess the ability of RBM25 to bind G4s *in cellulo*, we performed an additional PLA using an antibody raised against G4 (BG4, (63)) together with the antibody against RBM25 (Supplementary Figure 4). We observed numerous PLA dots, which are mostly localized in the nucleus (median value of 40 dots per nucleus as compared to one dot per nucleus as a median value for the negative control), clearly showing that RBM25 binds G4s in the cell, mostly in the nucleus.

Taken together, all these results indicate that RBM25 selectively binds in a G4-dependent manner to GQ-2, the rG4 that forms at the vicinity of the 5′ss leading to the short alternative pro-apoptotic Bcl-xS isoform (xS 5′ss).

### GQ-2 rG4 is required for both RBM25 binding and the synthesis of the alternative pro-apoptotic Bcl-xS isoform

**Figure 3.**
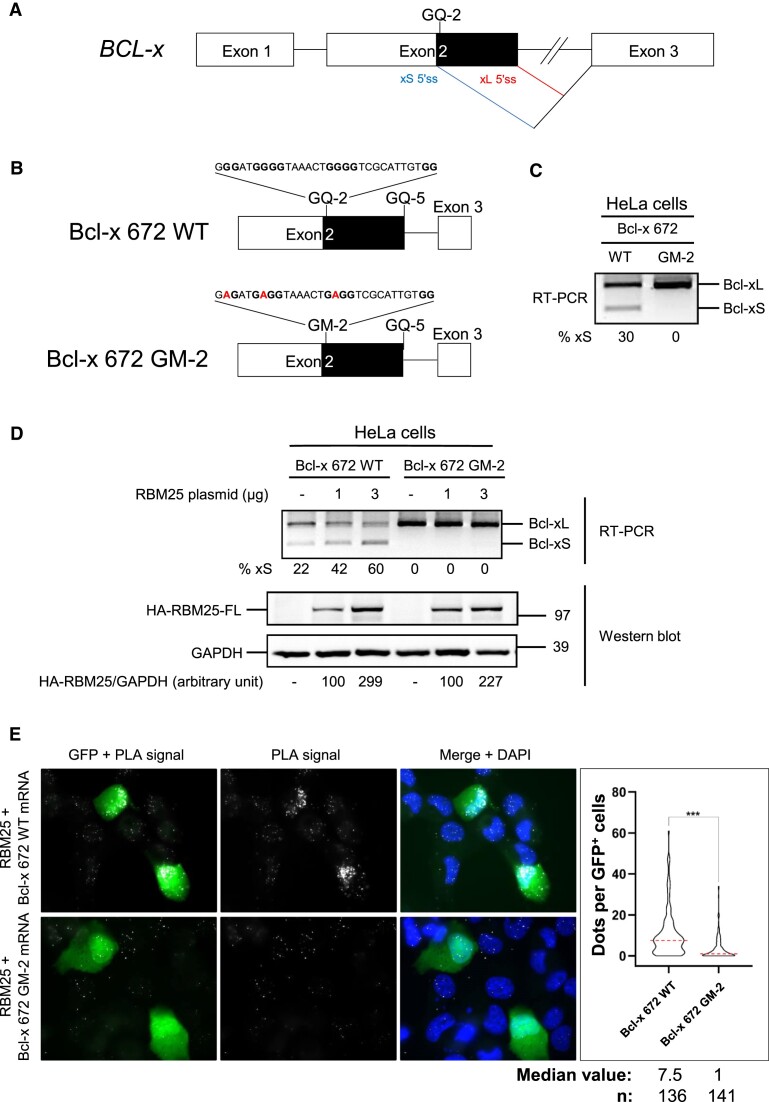
GQ-2 rG4 is required for both RBM25 binding and synthesis of the alternative pro-apoptotic Bcl-xS isoform (**A**) Schematic representation of the *BCL-x* gene with the two alternative 5′ splice sites in the exon 2 indicated (xS 5′ss in blue and xL 5′ss in red) as well as the GQ-2 rG4. (**B**) Schematic representation of the Bcl-x 672 WT (upper panel) and Bcl-x 672 GM-2 (lower panel) minigenes. The only differences between the WT and the GM-2 minigenes are the replacement of three guanines critical for GQ-2 rG4 formation by three adenines (highlighted in red). (**C**) Semi-quantitative RT–PCR experiment from HeLa cells transfected by the WT or GM-2 minigene, as indicated, to determine the relative proportion of the Bcl-xS and Bcl-xL isoforms synthesized from the minigenes. The PCR products were analysed by electrophoresis in a 1% agarose gel, revealed by an ethidium bromide staining and quantified by densitometry. An image of a resulting gel is shown (*n*≥ 3). (**D**) Same semi-quantitative RT–PCR experiment as in (**C**) except that, in addition to the WT or GM-2 minigene, HeLa cells were also transfected by the indicated quantities of a plasmid allowing expression of HA-tagged RBM25 wt (HA-RBM25-FL) or, as a control, by an empty plasmid. The result of the semi-quantitative RT–PCR experiment is shown in the upper panel and western blot analysis of the level of expression of HA-RBM25-FL, as compared to the loading control GAPDH, is shown in the two lower panels (*n*≥ 3). (**E**) Same PLA experiments as in Figure 2E, except that H1299 cells were transfected by a GFP-encoding plasmid that also express the WT or the GM-2 minigene, as indicated. Microscopy images of cells analysed using a probe specifically hybridizing to the *BCL-x* RNA transcribed from the minigenes. GFP signal indicates cells that were efficiently transfected and nuclei were revealed by DAPI staining and appear in blue. White dots (PLA signals) indicated interaction (close proximity) between the RBM25 protein and the *BCL-x* RNA transcribed from the minigene. The graph on the right indicates the number of PLA dots per cells in each condition. Data from two biological replicates, at least 35 GFP^+^ cells per sample, were analysed by a non-parametric Mann–Whitney's test using the GraphPad Prism 8 Software (***P< 0.0001).

We next designed two minigenes to monitor the alternative splicing of *BCL-x* and the effect of suppressing the ability of GQ-2 to form an rG4 structure on synthesis of the alternative product Bcl-xS and on the ability of RBM25 to interact with *BCL-x* RNA. First, based on an already published minigene (34,64,65), we constructed Bcl-x 672 WT (wild-type), a wt minigene that encompasses most of exon 2 (80% including all its 3′ part), part of intron 2 and the beginning of exon 3 (Figure 3A and B). Then, using site-directed mutagenesis we created a mutated version of this *WT* minigene (termed Bcl-x 672 GM-2, Figure 3B) by introducing the same modifications as in GM-2 (Figure 1B) making it unable of G4 formation (Figure 1C). We first assessed, using semi-quantitative RT–PCR, the alternative splicing from these two minigenes transfected in HeLa cells. As shown in Figure 3C, Bcl-x 672 WT led to ∼30% of Bcl-xS and ∼70% of Bcl-xL, in line with previously published results (34). By contrast, the Bcl-x 672 GM-2 minigene exclusively led to the synthesis of the canonical full length Bcl-xL isoform, readily suggesting that GQ-2 rG4 is important for alternative splicing of *BCL-x* and the synthesis of the alternative short pro-apoptotic Bcl-xS isoform.

Next, to further evaluate the validity of our minigene and the role of RBM25 binding on the GQ-2 rG4 in the production of the RNA encoding the alternative Bcl-xS isoform, we tested the effect of RBM25 overexpression on alternative splicing from either Bcl-x 672 WT or Bcl-x 672 GM-2 minigene. For this purpose, we transfected HeLa cells with both a plasmid that allows expression of Bcl-x 672 WT or Bcl-x 672 GM-2 minigene and increasing quantities (1 or 3 μg) of a plasmid allowing ectopic expression of a N-terminally HA-tagged version of RBM25 (HA-RBM25-FL). As shown in Figure 3D, the fraction of Bcl-xS RNA produced from the Bcl-x 672 WT minigene increased from 22 to 42% (with 1 μg of HA-RBM25-FL plasmid) and 60% (with 3 μg of HA-RBM25-FL plasmid), in good correlation with the increase in RBM25 (as assessed by comparing to the GAPDH loading control) and in line with previously published data (34,48). By contrast, irrespective of the level of RBM25, no Bcl-xS RNA was detected from the Bcl-x 672 GM-2 minigene, suggesting that the GQ-2 rG4/RBM25 interaction is required for synthesis of the alternative Bcl-xS isoform. To further test this possibility, we adapted the PLA shown in Figure 2E to specifically assess the interaction between endogenously expressed RBM25 and pre-mRNA expressed from either Bcl-x 672 WT or Bcl-x 672 GM-2 minigene transfected in H1299 cells. GFP was used as a marker to detect transfected cells. As shown in Figure 3E, the nuclei of GFP^+^ cells transfected by the Bcl-x 672 WT minigene contained multiple PLA dots (median value of 7.5 dots per cell) whereas the nuclei of the non-transfected GFP^−^ cells did not. By contrast, the nuclei of GFP^+^ cells transfected by the Bcl-x 672 GM-2 minigene contained only a few dots (median value of 1 dot per cell), which in addition were less intense and similar to the dots detected in non-transfected GFP^−^ cells. Additional controls are shown in Supplementary Figure 5. Altogether, these results further confirm that RBM25 interacts with GQ-2 rG4 present in the *BCL-x* pre-mRNA and that this interaction is necessary for the production of the alternative Bcl-xS isoform.

### The RE domain of RBM25 is necessary for binding to GQ-2 rG4 and defines a new rG4-binding motif

**Figure 4.**
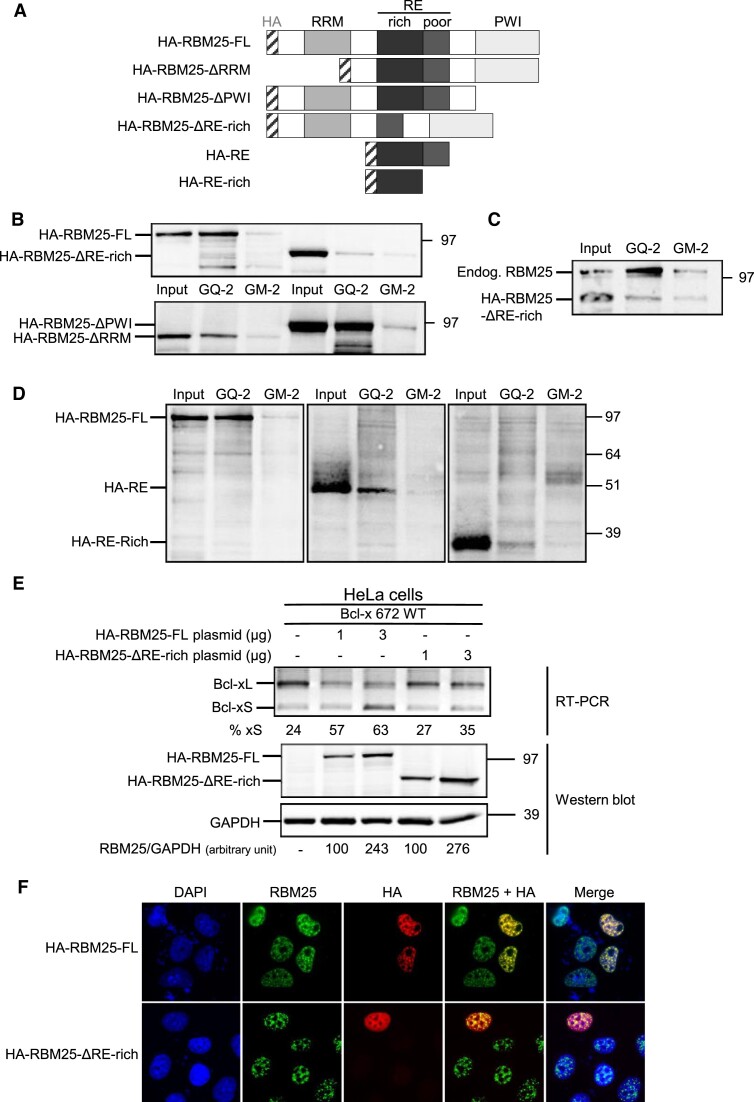
The RE domain of RBM25 is necessary and sufficient for binding to GQ-2 rG4. (**A**) Schematic representation of the RBM25 protein with its three motifs known to potentially interact with nucleic acids (RRM, RE and PWI) and of the various constructions used for the determination of the role of each of these three domains in binding to GQ-2 rG4. All the constructs are N-terminally HA-tagged (HA tag written in grey and represented as a hatched box). (**B**) Same RNA pulldown experiments as in Figure 2B using the GQ-2 or GM-2 matrices as indicated except that, in addition to H1299 cells transfected with an HA-tagged RBM25 (HA-RBM25-FL)-encoding plasmid, H1299 cells transfected with one or the other of the plasmids expressing the various HA-tagged forms of RBM25 deleted for one of its three domains were also used. The proteins still bound after an 800 mM KCl wash were eluted and analysed by SDS–PAGE and western blot using an anti-HA antibody to reveal the exogenously expressed various forms of HA-tagged RBM25. Gels represent *n*≥ 3. (**C**) Same experiment as in (**B**) using only the HA-RBM25-ΔRE-rich plasmid except that an anti-RBM25 antibody was used for the western blot analysis to reveal both exogenously expressed HA-RBM25-ΔRE-rich and endogenously expressed RBM25 that thus serves as an internal positive control. (**D**) Same experiment as in (**B**) except that a plasmid allowing expression of a HA-tagged full length RE motif (HA-RE) or only the RE-rich part of RE motif (HA-RE-rich) was used. (**E**) Same experiment as in Figure 3D except that, in addition to the effect of overexpressing HA-RBM25-FL on alternative splicing from the Bcl-x 672 WT minigene, the effect of overexpressing HA-RBM25-ΔRE-rich was also assessed. The result of the semi-quantitative RT–PCR experiment is shown in the upper panel and the western blot analysis of the level of expression of HA-RBM25-FL or HA-RBM25-ΔRE-rich, as compared to the loading control GAPDH, is shown in the two lower panels. Gels represent *n*≥ 3. (**F**) Immunofluorescence analysis of H1299 cells expressing HA-RBM25-FL or HA-RBM25-ΔRE-rich, as indicated. Microscopy images of fixed cells analysed using anti-RBM25 or anti-HA antibodies were shown as indicated. Merged images were also shown.

We next sought to identify which domain(s) of RBM25 is involved in its ability to interact with the GQ-2 rG4 of *BCL-x* pre-mRNA. As shown in Figure 4A, RBM25 contains three domains that may potentially be involved in binding to nucleic acids: an N-terminal RRM (RNA recognition motif) domain, a central RE (arginine-glutamate-rich) motif and a C-terminal PWI domain. To determine which of these three domains is important for binding of RBM25 to GQ-2 G4, we overexpressed HA-tagged versions of RBM25 deleted, or not, for one of the other of these three domains in H1299 cells (hence they are respectively termed HA-RBM25-ΔRRM, HA-RBM25-ΔPWI, HA-RBM25-ΔRE-rich and HA-RBM25-FL, depicted in Figure 4A) and then tested their ability to interact with GQ-2 G4 using the same RNA pulldown assay used in Figure 2A to C. Of note, the RE domain is composed of two sub-domains: one that is RE-rich, whereas the other is significantly less enriched in RE (termed RE-poor in Figure 4A). To minimize the size of the deletion, we choose to delete only the RE-rich part, hence the name of the construct: HA-RBM25-ΔRE-rich. As shown in Figure 4B, whereas HA-RBM25-FL, HA-RBM25-ΔPWI and HA-RBM25-ΔRRM interact in a G4-dependent manner with GQ-2 G4, only a residual binding barely superior to the residual binding on control GM-2 matrix was observed for HA-RBM25-ΔRE-rich. To confirm this result, we repeated this RNA pulldown experiment using protein extracts from H1299 cells transfected with HA-RBM25-ΔRE-rich and used an anti-RBM25 antibody to detect both endogenous and overexpressed HA-tagged RBM25. As shown in Figure 4C, endogenous RBM25 was clearly enriched on the GQ-2 matrix and only a residual binding was observed on the control GM-2 matrix, whereas only a residual binding of HA-RBM25-ΔRE-rich occurred on the GQ-2 matrix, which was barely superior to the one observed on the control GM-2 matrix. These results indicate that the RE motif is important for the ability of RBM25 to bind GQ-2 rG4 and readily suggests that the RE domain is directly mediating this interaction. To test this possibility, we expressed the HA-tagged RE motif (HA-RE), the RE-rich part only (HA-RE-rich) or HA-RBM25-FL (as a positive control) in H1299 cells and determined their ability to bind to the GQ-2 matrix compared to the control GM-2 matrix. As shown in Figure 4D, HA-RE is able to bind to GQ-2 in a G4-dependent manner, confirming that the RE motif is directly mediating the interaction of RBM25 with GQ-2 rG4. As for the HA-RE-rich construct, perhaps because it is most probably an intrinsically disordered motif, only a weak binding to GQ-2 RNA oligonucleotide was observed. However, even this weak binding was G4-dependent as no significant binding was observed with GM-2 oligonucleotide. Altogether, these results suggest that RE motif represents a new rG4-binding domain.

Next, we determined the effect of overexpressing HA-RBM25ΔRE-rich, as compared to HA-RBM25-FL, on splicing from the Bcl-x 672 WT minigene, using similar experiments as in Figure 3D. We observed that, although expressed at a level similar to HA-RBM25-FL (as determined by comparison to the GAPDH loading control, lower panels of Figure 4E), overexpression of HA-RBM25-ΔRE-rich only had a modest effect on alternative splicing (the proportion of Bcl-xS increasing from 24% in the negative control transfected by an empty plasmid to 27 and 35% in cells transfected with 1 or 3 μg, respectively, of the HA-RBM25-ΔRE-rich plasmid), which is in line with its limited ability to interact with GQ-2 rG4, as compared to the strong effect of HA-RBM25-FL (for which the proportion of Bcl-xS increased from 24% in the negative control to 57 and 63% in cells transfected with 1 or 3 μg, respectively, of the HA-RBM25-FL plasmid) (Figure 4E, upper panel). We have also determined the effect of overexpressing only HA-RE on splicing from the Bcl-x 672 WT minigene and found it had no effect (Supplementary Figure 6), suggesting that other domains of RBM25 are required for an effect on alternative splicing, most probably by recruiting various splicing factors.

Because the altered ability of HA-RBM25-ΔRE-rich to induce alternative splicing of RBM25 could be due to a change in its intracellular localization, we determined, using immunofluorescence, the localization of both HA-RBM25-FL and HA-RBM25-ΔRE expressed in H1299 cells. As shown in Figure 4F, we found a similar nuclear localization for both HA-RBM25-ΔRE and HA-RBM25-FL, in line with already published results (48). Hence, deleting the RE motif of RBM25, in addition to interfering with its capacity to interact with GQ-2 rG4 in *BCL-x* mRNA, also strongly affects its ability to induce alternative splicing of *BCL-*x. We conclude that interfering with the ability of RBM25 to interact with GQ-2 rG4 by deleting its RE domain has the same effect as interfering with this interaction by preventing rG4 formation through replacement of the three guanines critical for G4 formation in GQ-2 with adenines. Altogether, these results clearly indicate that the RE-mediated interaction between RBM25 and GQ-2 rG4 of *BCL-x* pre-mRNA is critical for the synthesis of the alternative pro-apoptotic Bcl-xS isoform.

### Effect of the G4 ligand PhenDC3 on RBM25/*BCL-x* RNA interaction and the synthesis of the alternative Bcl-xS isoform

**Figure 5.**
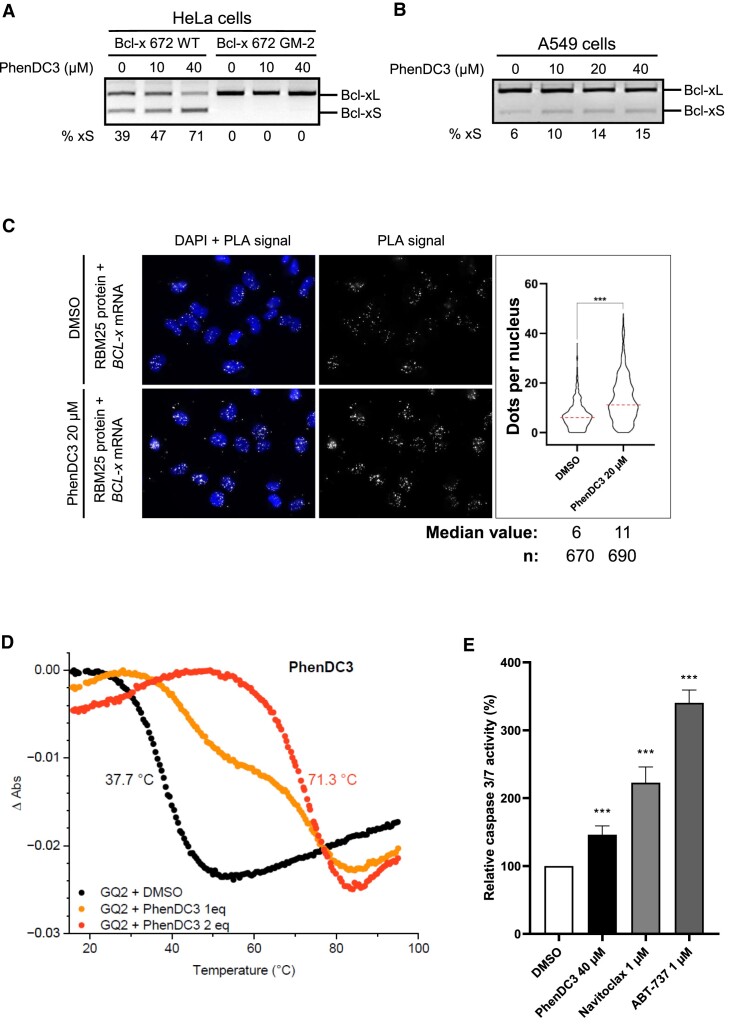
Effect of the PhenDC3 G4 ligand on RBM25/*BCL-x* RNA interaction, synthesis of the alternative pro-apoptotic Bcl-xS isoform and apoptosis. (**A**) Same semi-quantitative RT–PCR experiment as in Figure 3C to determine the relative proportion of the Bcl-xS and Bcl-xL isoforms synthesized from the minigenes, except that HeLa cells transfected by the WT or GM-2 minigene (as indicated) were also treated, or not, by various concentrations of the benchmark G4 ligand PhenDC3. Gel represents *n*≥ 3. (**B**) Same experiment as in (**A**) except that the alternative splicing of the endogenous *BCL-x* gene was analysed in A549 cells. Gel represents *n*≥ 3. (**C**) Same PLA experiment as in Figure 2E except that cells were treated, or not, with 20 μM PhenDC3. Microscopy images of H1299 cells analysed using a probe specifically hybridizing to the *BCL-x* RNA. Nuclei were revealed by DAPI staining and appear in blue. White dots (PLA signals) indicated interaction (close proximity) between endogenous RBM25 protein and endogenous *BCL-x* RNA. The graph on the right indicates the number of PLA dots per cells in each condition. Data from three biological replicates, at least 200 cells per sample, were analysed by a non-parametric Mann–Whitney's test using the GraphPad Prism 8 Software (***P< 0.0001). (**D**) Normalized UV-melting curves of GQ-2 (4 μM in lithium cacodylate buffer supplemented with 1 mM KCl and 99 mM LiCl) in the absence (black) or in the presence of 1 molar equivalent (orange) of 2 molar equivalents (red) of PhenDC3. (**E**) Determination, using the *Caspase Glo 3/7 Assay*, of the relative caspase 3/7 activity in A549 cells treated with DMSO (vehicle, negative control), 40 μM PhenDC3 or 1 μM Navitoclax or 1 μM ABT-737 as positive controls. Data from four biological replicates were analysed by ANOVA in conjunction with Tukey's test using GraphPad Prism 8 Software (***P< 0.0001).

Given the importance of GQ-2 rG4 on *BCL-x* alternative splicing, we next tested the effect of PhenDC3, a benchmark G4 ligand (66), on the splicing from the Bcl-x 672 WT minigene transfected into HeLa cells. As shown in Figure 5A, treatment with increasing concentration of PhenDC3 (from 0 to 40 μM) led to a dose-dependent increase (from 39 to 71%) in the proportion of the alternative Bcl-xS isoform while having no effect on Bcl-x 672 GM-2 minigene for which no alternative splicing was observed with or without PhenDC3.

To assess whether this effect was also observed for the endogenous *BCL-x* gene, we tested the effect of PhenDC3 on the alternative splicing of *BCL-x* in A549 cells. In this cell line, as in other cancer-derived cell lines, the level of alternative Bcl-xS is barely detectable using RT–PCR and represents only ∼6% of total Bcl-x in this experiment. However, increasing concentrations of PhenDC3 (from 0 to 40 μM) led to a dose-dependent increase in the alternative Bcl-xS RNA that reached ∼15% at 40 μM PhenDC3 (Figure 5B), further confirming that PhenDC3 induces the alternative splicing of *BCL-x* and suggesting that this G4 ligand favours RBM25 binding to GQ-2 rG4 and thereby the production of the alternative pro-apoptotic Bcl-xS isoform. To test this hypothesis, using similar PLA experiments as in Figure 2E, we determined the effect of PhenDC3 on the interaction between endogenous RBM25 and endogenous *BCL-*x RNA in H1299 cells. We observed (Figure 5C) that a treatment with 20 μM PhenDC3 significantly increases the number of PLA dots (median value of 11 dots per cell, most of them being nuclear, as compared to a median value of six dots per cell for the DMSO-treated cells). We concluded that the G4 ligand PhenDC3 favours the binding of RBM25 to GQ-2 rG4 of *BCL-x* RNA, readily explaining its ability to boost alternative splicing of *BCL-x*.

We then tested the effect of PhenDC3 on the stability of GQ-2 RNA G4. For this purpose, we performed UV-melting experiments. In a buffer containing 1 mM K^+^, the addition of 1 or 2 molar equivalents of PhenDC3 resulted in an increase of melting temperature of GQ-2 from 37.4 to 71.3°C (Δ*T*_m_ = 33.9°C, Figure 5D) with 2 molar equivalents giving evidence of a strong stabilizing effect. In buffers with higher K^+^ content (10 or 100 mM) the ligand-induced stabilization prevented the melting of G4 at temperatures below 95°C, prohibiting the precise determination of *T*_m_ values. This behaviour is in line with the known capacity of PhenDC3 to strongly stabilize various DNA and RNA G4 (66–69). Hence, it is likely that PhenDC3 also stabilizes GQ-2 rG4 in native *Bcl-x* mRNA, or promotes the formation of an rG4 at the expense of alternative secondary structures, thereby favouring RBM25 binding and consequently production of the alternative pro-apoptotic Bcl-xS isoform.

For this reason, we tested the effect of PhenDC3 on the induction of apoptosis in A549 cells only expressing endogenous Bcl-x. Using the *Caspase Glo^®^ 3/7 Assay* which allows to determine the relative caspase 3/7 activity, and using the BH3-mimetics Navitoclax and ABT-737 (70,71) as positive controls, we found that PhenDC3, in line with its ability to induce Bcl-xS, does induce apoptosis (Figure 5E), as compared to the negative control (DMSO 0.4%; compound vehicle).

### Screening of a library of 90 putative G4 ligands leads to the identification of two original and more active modulators of *BCL-x* splicing

**Figure 6.**
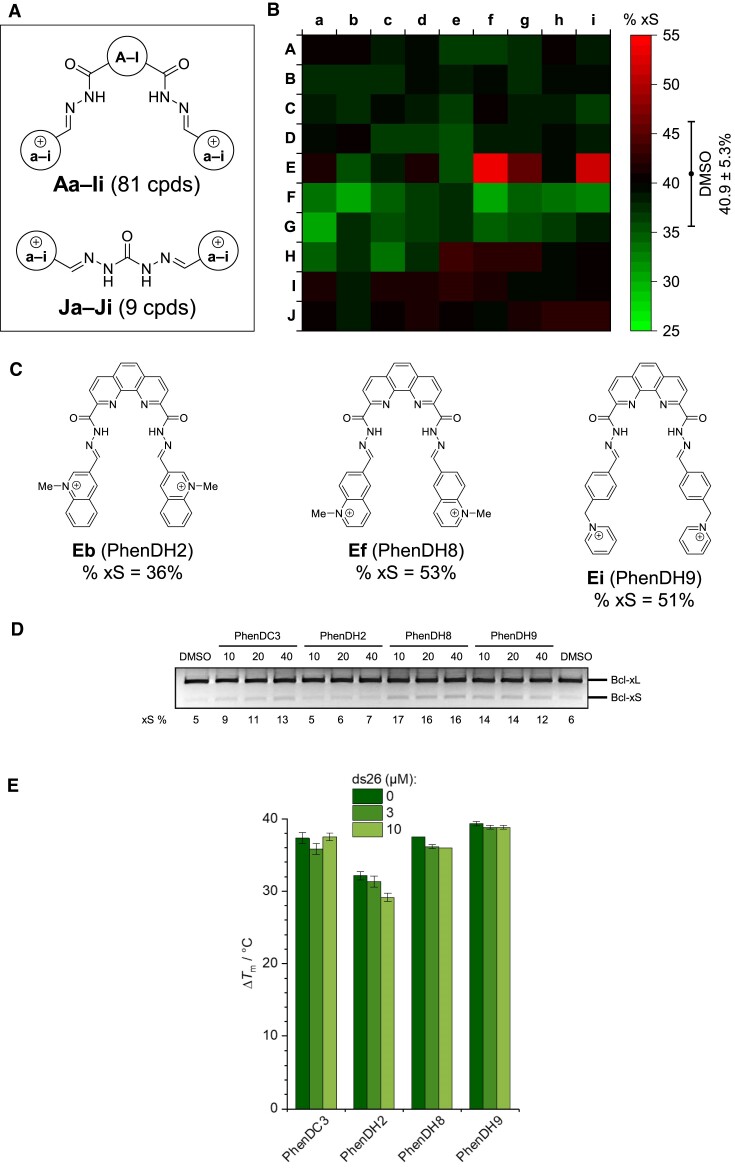
Screening of a library of 90 putative G4 ligands and identification of PhenDH2, 8 and 9. (**A**) Generic structure of the combinatorial library of 90 cationic bis(acylhydrazones) (**Aa**–**Ji**). (**B**) The effect of individual library members (at ∼10 μM) on alternative splicing from the Bcl-x 672 WT minigene, as determined by semi-quantitative RT–PCR experiments on HeLa cells transfected by the Bcl-x 672 WT minigene was determined. The percentage of Bcl-xS obtained for each compound is indicated as a heatmap (% xS). PhenDC3 at the same concentration was used as a positive control. (**C**) Chemical structures of hit compounds **Ef** (PhenDH8), **Ei** (PhenDH9) and the inactive analogue **Eb** (PhenDH2). (**D**) Semi-quantitative RT–PCR experiments to assess alternative splicing of the endogenous *BCL-x* gene in A549 cells submitted to increasing concentrations of PhenDC3, PhenDH8, PhenDH9 or PhenDH2, as indicated. PhenDH8 and 9 are more potent than PhenDC3 to induce the alternative pro-apoptotic Bcl-xS whereas PhenDH2, although structurally close, is inactive at all tested concentrations. Gel represents *n*≥ 3. (**E**) Ligand-induced stabilization of GQ2 observed in FRET-melting experiments, performed with double-labelled oligonucleotide (F-GQ2-T, 0.2 μM) in the presence of the ligands (1.0 μM each) and in the absence or in the presence of double-stranded DNA competitor (ds26: 0, 3 or 10 μM). Experiments were performed in 10 mM lithium cacodylate buffer (pH 7.2) supplemented with 10 mM KCl and 90 mM LiCl.

The ability of PhenDC3 to enhance the binding of RBM25 to rG4 in *BCL-x* pre-mRNA, and thereby to promote the alternative pro-apoptotic Bcl-xS isoform and apoptosis indicates that favouring the interaction between RBM25 and the GQ-2 rG4 in *BCL-x* pre-mRNA represents a relevant intervention point to re-sensitize cancer cells to chemotherapy. Hence, we screened a combinatorial library of 90 ‘as-synthesized’ cationic bis(acylhydrazones) **Aa-Ji** (Figure 6A and Supplementary Figure 7), structurally related to PhenDC3 and previously validated as putative G4 ligands (50) using a fixed concentration of ∼10 μM and the splicing from the Bcl-x 672 WT minigene transfected into HeLa cells as a readout (Figure 6B and Supplementary Figure 8). From these 90 compounds, two derivatives containing a phenanthroline fragment (chemical structures: Figure 6C) significantly increased the Bcl-xS ratio (**Ef**, or PhenDH8: 53% xS; **Ei**, or PhenDH9: 51% xS). Of note, several structurally close phenanthroline derivatives, such as **Eb** (PhenDH2), were inactive. We then tested pure samples of PhenDH8, PhenDH9, PhenDC3 and PhenDH2 (as inactive control) at various concentrations (from 0 to 40 μM) to rank them in terms of efficacy. We found the following order of potency: PhenDH8 > PhenDH9 > PhenDC3 > PhenDH2, the latter being only weakly active at the highest concentration tested (40 μM), whereas PhenDH8 was already fully active at the lowest concentration (10 μM) (Supplementary Figure 9). Next, we tested the effect of these four compounds on the alternative splicing of the endogenous *BCL-x* gene in A549 cells. As shown in Figure 6D, we obtained similar results than when using the minigene, with the same order of potency, confirming that the Bcl-x 672 WT minigene is a relevant tool to model *BCL-x* alternative splicing.

To rationalize the results obtained with these compounds, we assessed their capacity to stabilize the rG4 structure formed by GQ-2 by FRET-melting experiments. The results (Figure 6E) indicated that PhenDH8 was as effective as PhenDC3 in stabilizing rG4 (Δ*T*_m_ = 37.5°C), PhenDH9 was slightly more effective (Δ*T*_m_ = 39.3°C), and PhenDH2 significantly less effective than PhenDC3 (Δ*T*_m_ = 32.2°C). Moreover, and in contrast to PhenDC3, PhenDH8 and PhenDH9, the stabilization effect induced by PhenDH2 was further reduced when the experiment was performed in the presence of double-stranded DNA (ds26) as a competitor, indicating that in cellular conditions this ligand has poor capacity to stabilize rG4 in *BCL-x* pre-mRNA. Taken together, these experiments shed light on the relative capacity of the ligands to modulate *BCL-x* splicing.

**Figure 7.**
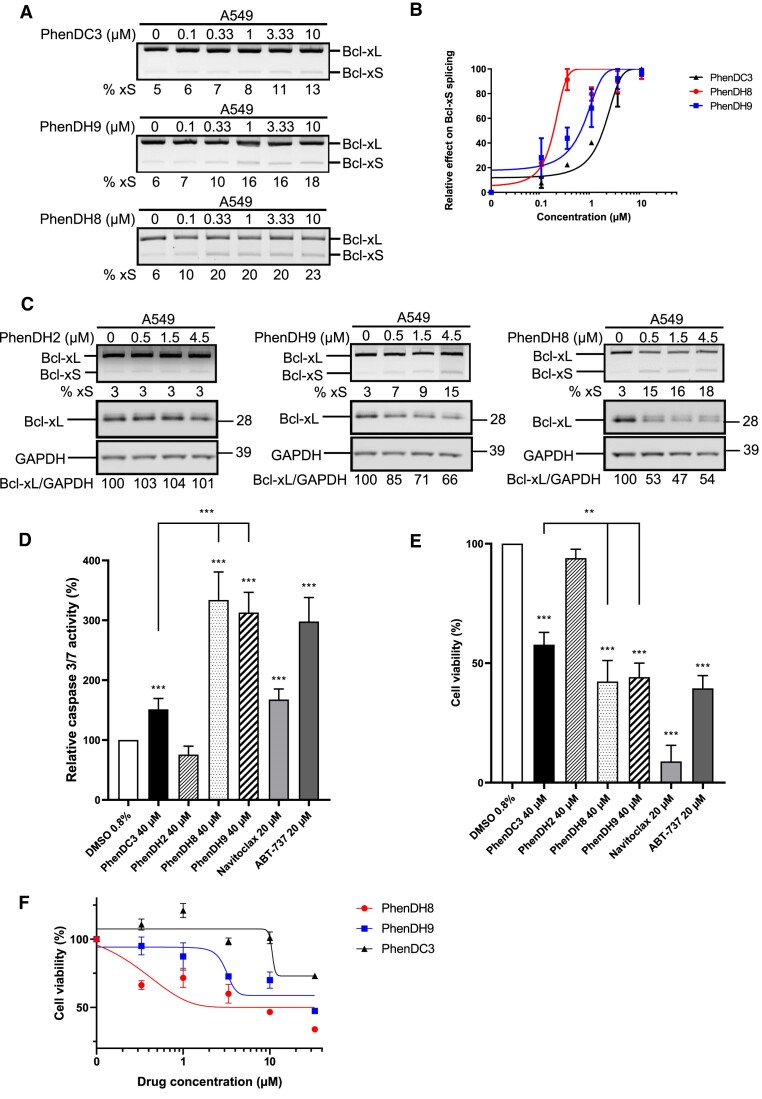
Quantitative analysis of the effect of PhenDC3, PhenDH8 and PhenDH9 on alternative splicing of *BCL-x*, apoptosis and cytotoxicity. (**A**) Semi-quantitative RT–PCR experiments to assess alternative splicing of the endogenous *BCL-x* gene in A549 cells submitted to increasing concentrations of PhenDC3, PhenDH9 or PhenDH8, as indicated. Gels represent *n*≥ 3. (**B**) Semi-logarithmic representation of the data shown in (**A**). (**C**) Western blot analysis of the effect of a range of concentration of PhenDH2, PhenDH9 or PhenDH8 (as indicated) on the protein level of the anti-apoptotic isoform Bcl-xL (middle gels) as compared to GAPDH (loading control, lower gels). The impact of the various G4 ligands on the alternative splicing of the endogenous *BCL-x* gene was determined in the same samples using semi-quantitative RT–PCR experiments (upper gels) as in (**A**). Gels represent *n*≥ 3. (**D**) Effect of PhenDC3, PhenDH8, PhenDH9 or PhenDH2 (all at 40 μM) on apoptosis as determined using the *Caspase Glo 3/7 Assay* as in Figure 5E. DMSO (vehicle) was used as a negative control and Navitoclax and ABT-737 were used as positive controls. Data from three biological replicates were analysed by ANOVA in conjunction with Tukey's test (***P< 0.0001). (**E**) Effect of PhenDC3, PhenDH8, PhenDH9 or PhenDH2 (all at 40 μM) on cell viability as determined using the MTT assay. DMSO (vehicle) was used as a negative control and Navitoclax and ABT-737 were used as positive controls. Data from three biological replicates were analysed by ANOVA in conjunction with Tukey's test using GraphPad Prism 8 Software (**P< 0.001, ***P< 0.0001). (**F**) Semi-logarithmic representation of the data obtained when testing the effect of a range of concentrations of PhenDC3, PhenDH8, PhenDH9 or PhenDH2 on cell viability as determined using the MTT assay as in (**E**).

Next, we tested the effect of a range of lower concentrations of the various G4 ligands on alternative splicing of the endogenous *BCL-x* gene (Figure 7A). This allowed us to estimate the EC_50_ (half-maximum effective concentration) for the three active G4 ligands, thereby confirming their order of potency: PhenDH8 (EC_50_ ≈ 0.2 μM) > PhenDH9 (EC_50_ ≈ 0.7 μM) > PhenDC3 (EC_50_ ≈ 2 μM) (Figure 7B). Then, to determine whether the effect of these G4 ligands on alternative splicing of the endogenous *BCL-x* affects the anti-apoptotic Bcl-xL protein level, we performed western blots using an antibody specific for the Bcl-xL isoform. As shown in Figure 7C, PhenDH8 and PhenDH9 led to a significant decrease in the level of Bcl-xL protein, which is consistent with their effect on the alternative splicing of the *BCL-x* gene. By contrast, PhenDH2 had no effect on Bcl-xL level, in line with its lack of effect on splicing of *BCL-x*.

The ability of PhenDH8, PhenDH9 and PhenDC3 to affect the alternative splicing of *BCL-x* and to promote the alternative pro-apoptotic Bcl-xS isoform being confirmed, we then tested their ability to induce apoptosis, using PhenDH2 and DMSO (the vehicle) as negative controls and Navitoclax and ABT-737, two BH3-mimetics (70,71), as positive controls. For this purpose, we used the same Caspase Glo^®^ 3/7 Assay as in Figure 5E, which allows to determine the relative caspase 3/7 activity. As shown in Figure 7D, we first confirmed that PhenDC3 induces apoptosis. We also observed that PhenDH8 and PhenDH9 were even more active, whereas PhenDH2 was inactive. Importantly, we got the same order of potency than for the ability of the compounds to induce the synthesis of the alternative pro-apoptotic Bcl-xS isoform.

Finally, using the MTT assay, we measured the ability of the compounds to induce cell death (Figure 7E). We obtained the same result than in the two splicing assays and in the Caspase Glo^®^ 3/7 Assay. To determine whether the compounds have the same order of potency than on alternative splicing, we repeated the MTT assays with a range of concentrations for each of the three active G4 ligands (Figure 7F). This allowed us to estimate the GI_50_ (the concentration of the agent that inhibits growth by 50%) for each of these compounds. We found again that PhenDH8 was the most active compound, followed by PhenDH9 and then by PhenDC3.

Taken together, these results indicate that PhenDC3, a benchmark G4-ligand, and its two derivatives PhenDH8 and PhenDH9, induce both the alternative pro-apoptotic Bcl-xS isoform and apoptosis and that the intensity of their effect on apoptosis and cell death is correlated with their effect on alternative splicing.

## Discussion

In this paper, using RNA pulldown and PLA adapted for monitoring protein–RNA interactions, we first show that RBM25 binds directly and specifically to the GQ-2 rG4 of *BCL-*x RNA. We also show that this interaction is G4 structure-dependent and involves the central RE motif of RBM25, thereby defining a new G4-interacting domain. In addition, we found that this RBM25/GQ-2 rG4 interaction is necessary for alternative splicing of *BCL-x* and is a limiting factor for the synthesis of the pro-apoptotic Bcl-xS isoform. Indeed, interfering with this interaction, either by preventing G4 formation (GM-2 mutant) or by deleting the RE motif of RBM25, abolishes the synthesis of Bcl-xS, the pro-apoptotic alternatively spliced form of Bcl-x. On the contrary, favouring the RBM25/GQ-2 rG4 interaction by overexpressing RBM25 or by using PhenDC3, a G4 ligand that strongly stabilizes GQ-2 rG4 *in vitro*, promotes the synthesis of Bcl-xS and apoptosis. Importantly, this last result indicates that the RBM25/GQ-2 rG4 interaction represents an intervention point to modulate the balance between Bcl-xL and Bcl-xS, the two antagonistic forms of Bcl-x. This is of particular interest for the various disorders linked to overexpression of one or the other of Bcl-x isoforms, in particular for cancers in which overexpression of the anti-apoptotic Bcl-xL isoform has been associated to resistance to chemotherapy (15). Hence, small molecules that promote the pro-apoptotic alternative Bcl-xS isoform (such as PhenDC3, PhenDH8 and PhenDH9) may represent prototypes of candidate drugs to re-sensitize cancer cells to chemotherapy. Importantly, the fact that PhenDC3, PhenDH8 or PhenDH9 promote both the Bcl-xS isoform and apoptosis is not a proof of a mechanism in which they would induce apoptosis as a consequence of their effect on *BCL-x* alternative splicing as these two effects could be independent. However, the fact that the order of potency of these compounds on apoptosis (PhenDH8 > PhenDH9 > PhenDC3 > PhenDH2) is the same than on induction of the pro-apoptotic Bcl-xS isoform, and that PhenDH2, their close chemical derivative, is inactive on both processes suggest that a causal link exists between induction of Bcl-xS and of apoptosis by our active G4 ligands. Of note, novel molecular tools have recently been developed that rather destabilize G4 (72). According to the results presented here, these new compounds may thus represent therapeutic options to interfere with the production of the alternative pro-apoptotic Bcl-xS isoform whose overexpression has been linked to various disorders such as some forms of diabetes in which it has been associated to exacerbated apoptosis of β cells in the islets of Langerhans (15).

Important questions remain, such as the precise mechanism of action of RBM25 in *BCL-x* alternative splicing. At least two possible models could be envisioned. In the first model, the GQ-2 rG4 may represent a protein-binding platform recognized by the splicing factor RBM25 that itself might promote the selection of the xS 5′ss by the splicing machinery, thereby leading to the synthesis of the alternative pro-apoptotic Bcl-xS isoform. In the second model, the main role of RBM25 in *BCL-x* alternative splicing could be to promote and/or stabilize GQ-2 rG4 formation which would be required for the selection of the xS 5′ss by the splicing machinery. The first hypothesis is supported by the effects of G4 ligands such as PhenDC3, which are likely to increase the lifetime and/or the fraction of GQ-2 rG4 at the expense of alternative secondary RNA structures, thereby enhancing the binding of RBM25. At the same time, in favour of the second hypothesis, recent *in vitro* data (35) showed that the stabilizing effect of a G4 ligand (GQC-05, an ellipticin derivative) on GQ-2 rG4 could be observed only in the presence of nuclear extract. This suggests that the existence of this rG4 *in cellulo* requires the presence of a nuclear factor, which may be an RNA binding protein such as RBM25, that would promote its formation and thereby its recognition and further stabilization by a G4 ligand. Clearly, further investigations are required to decide between the two hypotheses.

Importantly, whatever the precise mechanism of action of RBM25 in promoting the alternative splicing of *BCL-x*, this mechanism represents an intervention point at which to interfere using notably G4 ligands (73,74). Indeed, several modes of action are possible for G4 ligands: they can either stabilize or destabilize G4, or they can prevent binding of a factor on G4, as shown for PhenDC3, PhenDH2 and PyDH2 that have been reported to compete with nucleolin for binding to rG4 that form in the Esptein–Barr virus-encoded EBNA1 mRNA and in the Kaposi's sarcoma-associated herpesvirus-encoded LANA1 mRNA, thereby interfering with immune evasion of these two oncoviruses (30,31,75).

An inherent limitation in the use of G4 ligands may be their potential lack of specificity given that, in addition to the numerous rG4, there are hundreds of thousands potential DNA G4 that may form in the human genome. Importantly, rG4 are more stable than DNA G4, in particular because rG4 predominantly adopt parallel structures, in contrast to DNA G4 that may fold into either parallel, antiparallel of hybrid conformations. In addition, due to the presence of the complementary strand, DNA G4 are in competition with the formation of DNA duplex by canonical Watson–Crick pairing. Hence, G4 ligands may preferentially target rG4. Also, RNA is inherently more flexible than DNA and may adopt a plethora of alternative conformations involving G4 as well as other structural features (76). Thus, G4 in the RNA context are often in a dynamic equilibrium with other structural isoforms, and even low doses of G4 ligand may have a profound impact on this equilibrium, preventing or leading to the recruitment of the corresponding structure-specific binding factors. Another important point is that, beside the G-quartets, which are the constant elements constitutive of G4, there are other structural characteristics, which vary from one sequence to another: the loops and the flanking regions. These elements ensure that each quadruplex is, in principle, unique within the whole genome and, as such, could be exploited as key targets to gain specificity through, for instance, oligonucleotide (or derivatives)-based approaches. Hence it is not excluded that some specificity toward particular G4 may be reached and various methods to assess G4 ligands specificity have been described (77,78). In line, the screening of various libraries of G4 ligands against some particular G4 clearly indicates that selectivity may be reached (34,52,78) and coupling oligonucleotides complementary to the loops or the flanking sequences to G4 ligands can direct them onto some specific G4 (79). Importantly, the specificity issue is also encountered in drug development programmes based on slicing factors or splicing kinases such as CLK or DYRK1A as the developed compounds may, in principle, act on the (alternative) splicing of thousands of genes.

Another important issue, when interfering with the Bcl-xL/Bcl-xS balance, is the risk of potential side effects, for example when promoting Bcl-xS, given its suspected role in various disorders such as some forms of diabetes or of cardiac disorders. Also, promoting Bcl-xL may favour tumour formation. Hence, the targeting of drugs interfering with the Bcl-xL/Bcl-xS balance to specific organs or to tumour cells is an important point to consider in future drug development programmes.

Finally, other means to modify the Bcl-xL/Bcl-xS balance have been developed, such as splice switching oligonucleotides (SSO), splicing kinases inhibitors or BH3-mimetics (15,70,71). Some of these drug candidates have shown promising effects in various preclinical or clinical trials, in particular BH3-mimetics, but their further development has been limited by their side effects, in particular for BH3-mimetics such as ABT-737 (80) and ABT-263 (Navitoclax, (81)). Hence, the possibility to develop the use of G4 ligands such as PhenDH8 or PhenDH9 in co-treatment with reduced quantities of these drug candidates may represent an appealing possibility to limit their side effects.

## Supplementary Material

gkad772_Supplemental_File

## Data Availability

The data underlyingthis article are either available in the article or in its online supplementary materials, or will be made available from the corresponding author upon reasonable request.
