# Supplementary material for: Alternative splicing of BCL-x is controlled by RBM25 binding to a G-quadruplex in BCL-x pre-mRNA
Source: Nucleic Acids Res. 2023 Oct 9;51(20):11239–57. doi: 10.1093/nar/gkad772 (PMC10639069; doi:10.1093/nar/gkad772)
Supplement: gkad772_Supplemental_File [file gkad772_supplemental_file.pdf]

Supplementary Figure 1

Oligonucleotide	Sequence (5′→3′)
GQ2	GGGAUGGGGUAAACUGGGGUCGCAUUGUGG
GM2	GAGAUGAGGUAAACUGAGGUCGCAUUGUGG
GQ2-A22	GGGAUGGGGUAAACUGGGGUCACAUUGUGG
GQ2-A27	GGGAUGGGGUAAACUGGGGUCGCAUUAUGG
GQ2-A22A27	GGGAUGGGGUAAACUGGGGUCACAUUAUGG

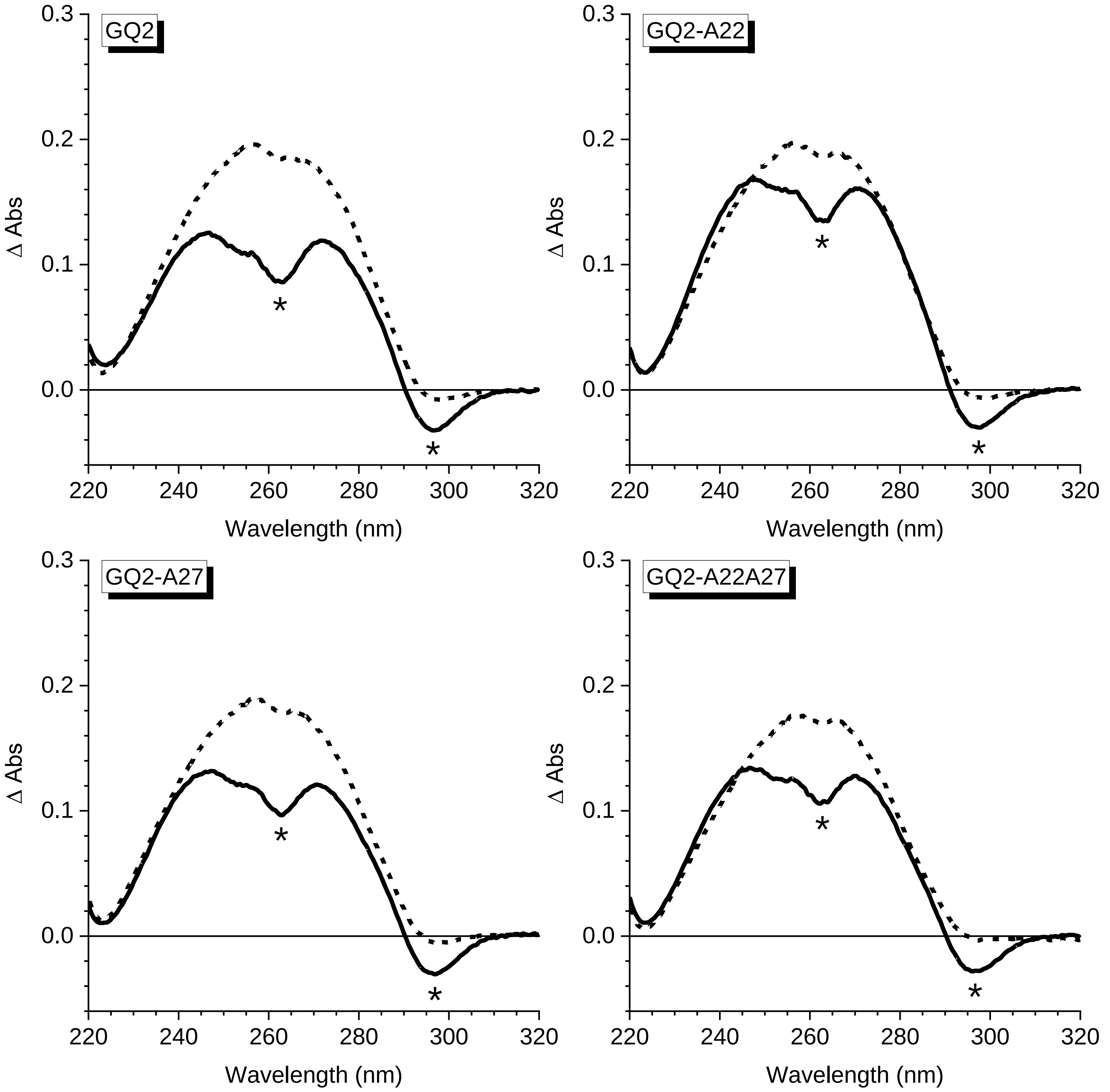

Sequences and thermal difference spectra (ΔAbs = $Abs_{80\,°C}$ − $Abs_{20\,°C}$) of GQ-2 and its variants. TDS were obtained with 4 µM oligonucleotides in 10 mM lithium cacodylate buffer (pH 7.2) supplemented with 100 mM KCl (solid lines) or 100 mM LiCl (dashed lines). G4-characteristic peaks are indicated with asterisks.

Supplementary Figure 2

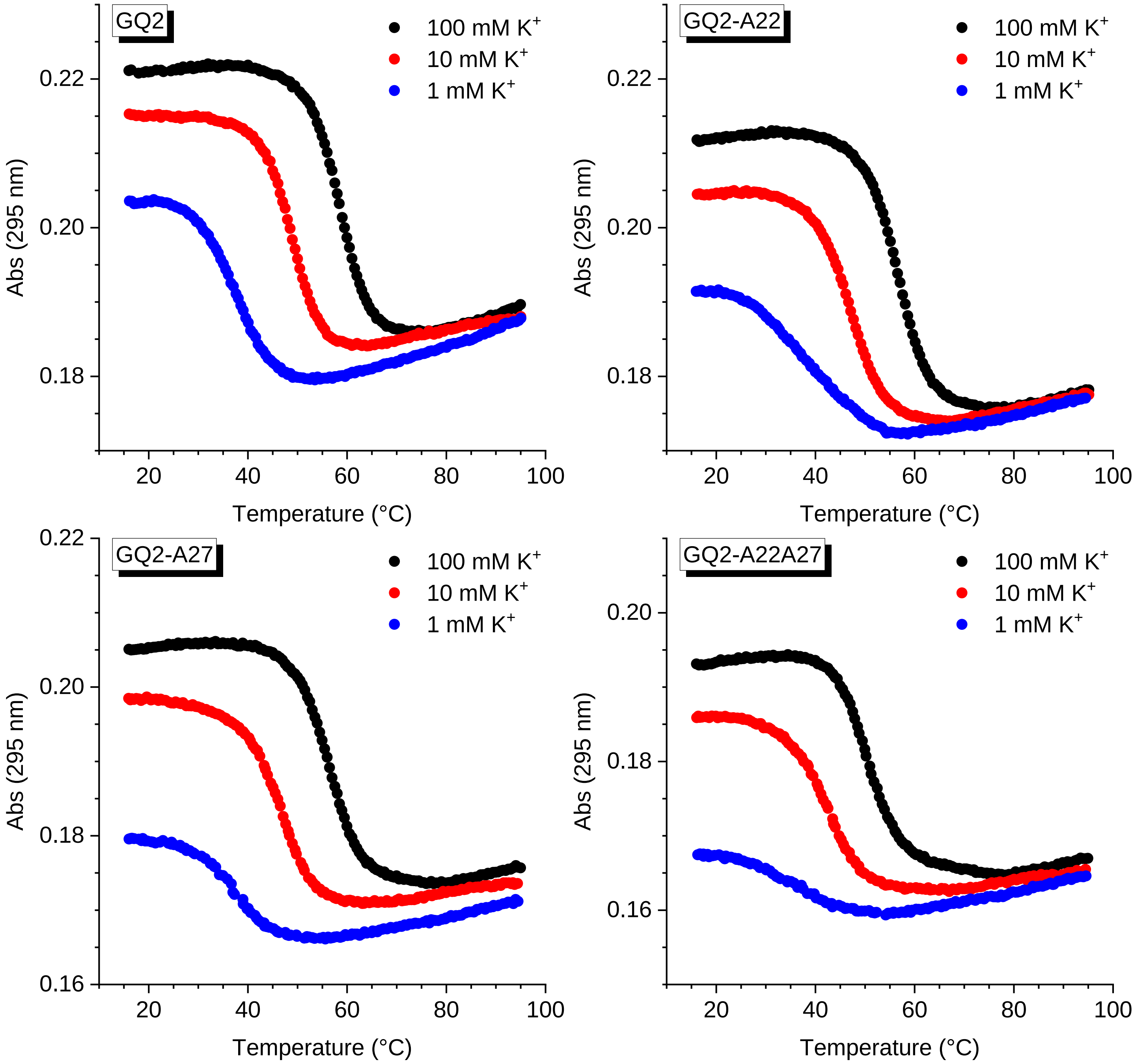

Oligo	T_m / °C, at K+ concentration (mM):		
	1	10	100
GQ2	37.4	48.8	58.6
GQ2-A22	38.5	47.1	56.6
GQ2-A27	36.6	46.9	58.4
GQ2-A22A27	35.6	42.8	50.4

UV-melting analysis of GQ-2 and its variants. UV melting profiles were obtained with 4 µM oligonucleotides in 10 mM lithium cacodylate buffer (pH 7.2) supplemented with various concentrations of KCl (as indicated) and LiCl to a total ionic strength of 110 mM.

Supplementary Figure 3

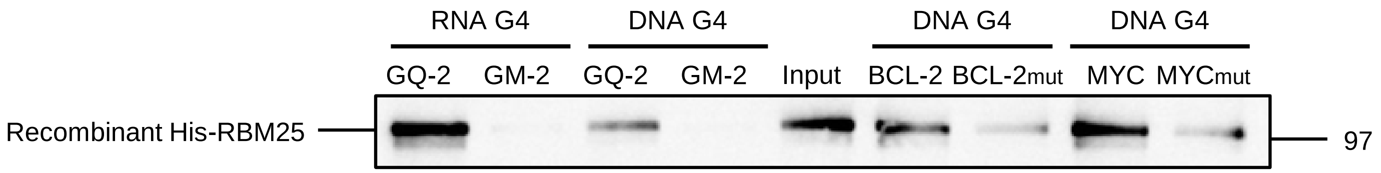

RNA or DNA pulldown (as indicated) of a recombinant polyhistidine-tagged RBM25 protein (His-RBM25) using the indicated RNA or DNA oligonucleotides matrices were performed as indicated in **Figure 2**. Recombinant RBM25 protein still bound after an 800 mM KCl wash was eluted and analysed by SDS-PAGE and western blot using and anti-RBM25 antibody. Three different DNA matrices were used: GQ-2 which has the same sequence than the GQ-2 RNA matrix used in **Figure 2** to compare the ability of RBM25 to bind to rG4 and dG4 of the same sequence, BCL-2 and MYC (with their mutated versions, respectively BCL-2mut and MYCmut in which a minimal number of guanines involved in G4 formation were replaced by adenines or thymidines to prevent G4 formation as predicted using the G4Killer software, Brazda V et al, *Bioinformatics* 2020).

The following G-quadruplex forming oligonucleotides (RNA or DNA as indicated) 3'-tagged with TEG-Biotin were used:
GQ-2 (RNA) GGGAUGGGGUAAACUGGGGUCGCAUUGUGG-3'-TEG-Biotin,
GM-2 (RNA) GAGAUGAGGUAAACUGAGGUCGCAUUGUGG-3'-TEG-Biotin,
GQ-2 (DNA) GGGATGGGGTAAACTGGGGTCGCATTGTGG-3'-TEG-Biotin,
GM-2 (DNA) GAGATGAGGTAAACTGAGGTCGCATTGTGG-3'-TEG-Biotin,
BCL-2 (DNA) AGGGGCGGGCGCGGGAGGAAGGGGGCGGGAGCGGGGCTG-3'-TEG-Biotin,
BCL-2mut (DNA) AGGTGCGGGCGCGTGAGGAAGGTGGCGAGAGCGGAGCTG -3'-TEG-Biotin,
MYC (DNA) TGGGGAGGGTGGGGAGGGTGGGGAAGG -3'-TEG-Biotin and
MYCmut (DNA) TGGAGAGAGTGGAGAGAGTGGTGAAGG-3'-TEG-Biotin.

Supplementary Figure 4

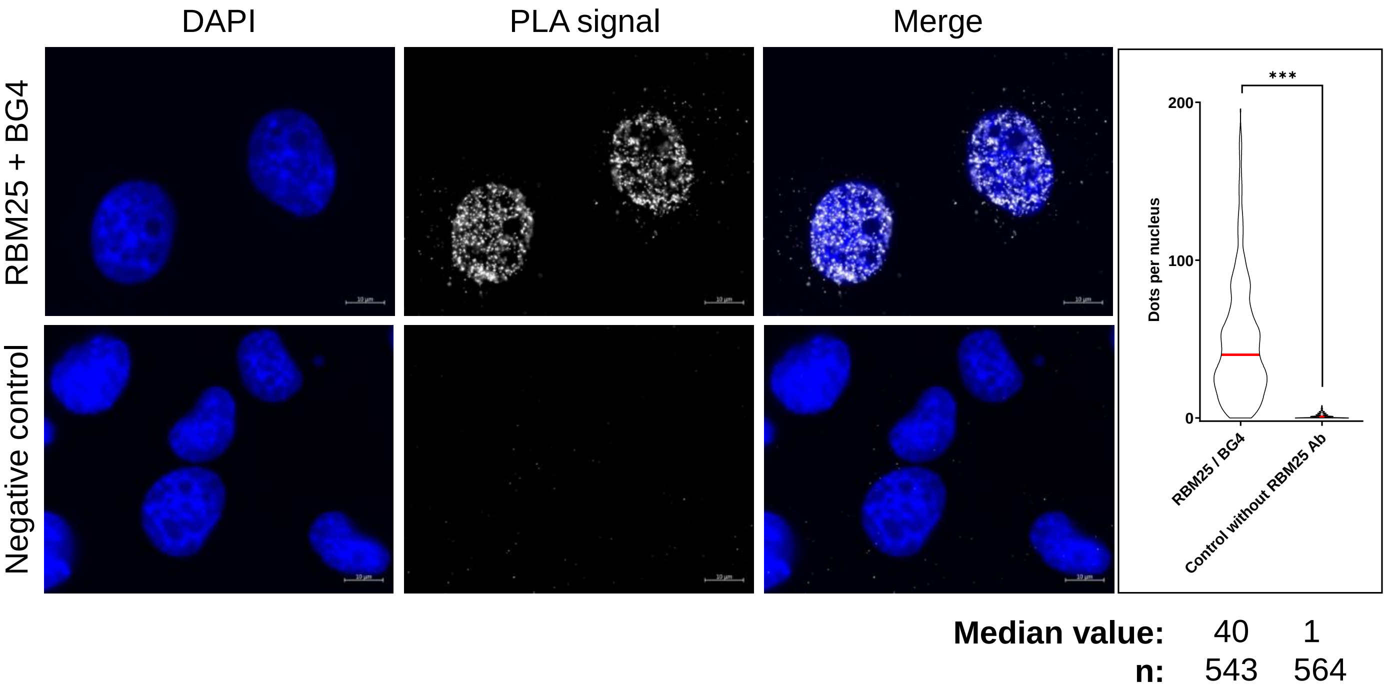

Proximity ligation assay (PLA) performed in H1299 cells to monitor *in cellulo* the interaction between endogenously expressed RBM25 protein and G4 using a BG4 antibody and an anti-RBM25 antibody. Microscopy images of H1299 cells analysed using both BG4 (anti-G4 antibody) and an anti-RBM25 antibody (upper panels) or only the BG4 antibody (lower panels, negative controls). Nuclei were revealed by DAPI staining and appear in blue. White dots (PLA signals) indicate interaction (close proximity) between RBM25 protein and G4 (that can be rG4 or dG4). The graph on the right indicates the number of PLA dots per cells in each condition. Data from three biological replicates, at least 500 cells per sample, were analysed by a non-parametric Mann-Whitney's test using the GraphPad Prism 8 Software (*** P<0.0001).

Supplementary Figure 5

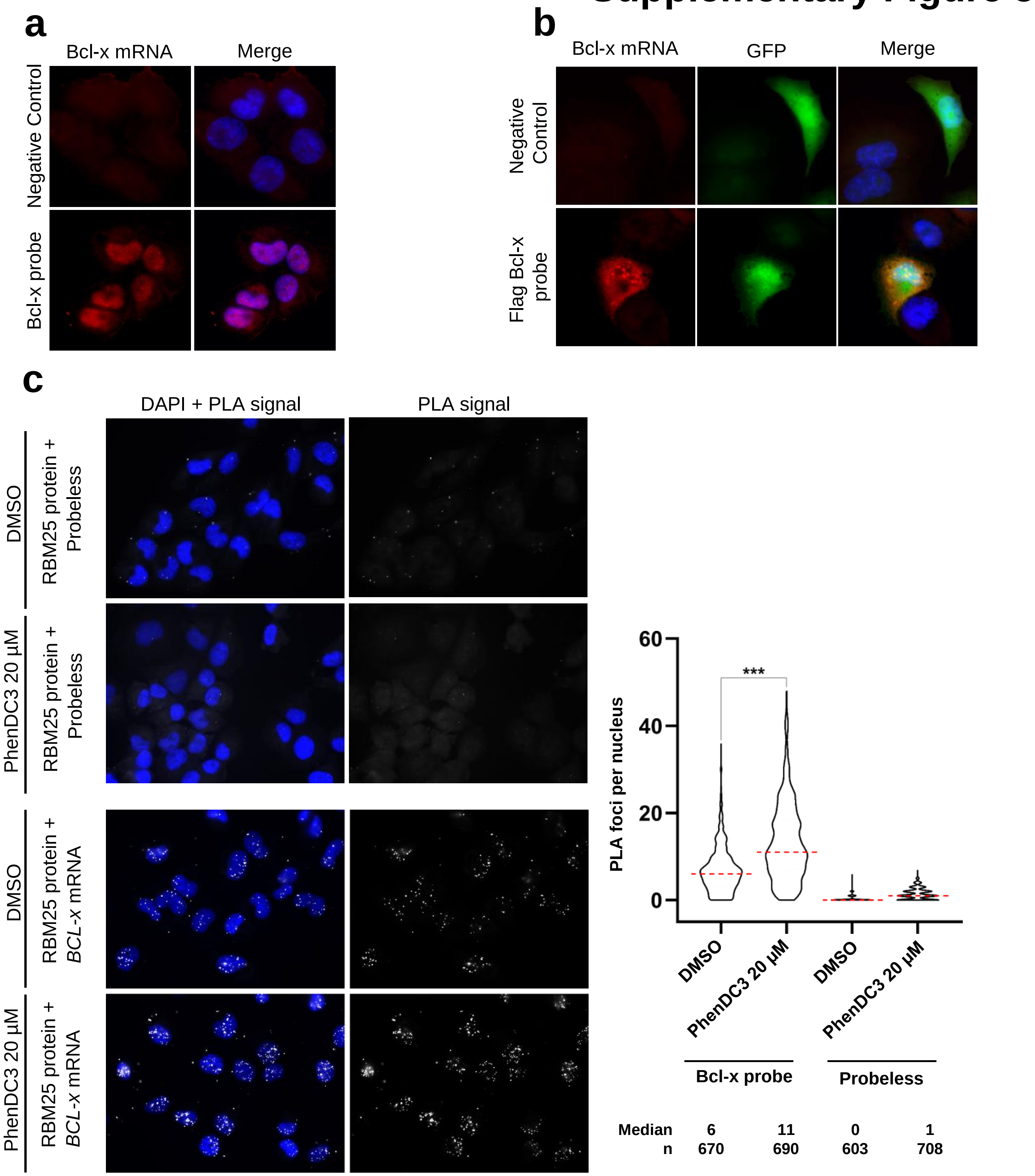

H1299 cells **(a)** and H1299 cells transfected with the minigene Bcl-x 672 WT **(b)** were analyzed by RNA *in situ* hybridization-immunofluorescence (rISH-IF) to verify the specificity of the Bcl-x digoxigenin probe **(a)** and the Flag Bcl-x digoxigenin probe **(b)** and to validate the detection of probe-mRNA complexes. **(c)** Adaptation of the proximity ligation assay (PLA) to monitor the RBM25 protein-*BCL-x* mRNA interaction performed in H1299 cells natively expressing RBM25 protein and *BCL-x* mRNA. Microscopy images of H1299 cells analysed using a probe specifically hybridizing to the *BCL-x* RNA (lower panels) or not (upper panels, negative controls) with or without PhenDC3 treatment as indicated. Nuclei were revealed by DAPI staining and appear in blue. White dots (PLA signals) indicated interaction (close proximity) between RBM25 protein and *BCL-x* RNA. The graph on the right indicates the number of PLA dots per cells in each condition. Data from three biological replicates, at least 250 cells per sample, were analysed by t-test in conjunction with Mann-Whitney's test using GraphPad Prism 8 Software (*** P<0.0001).

Supplementary Figure 6

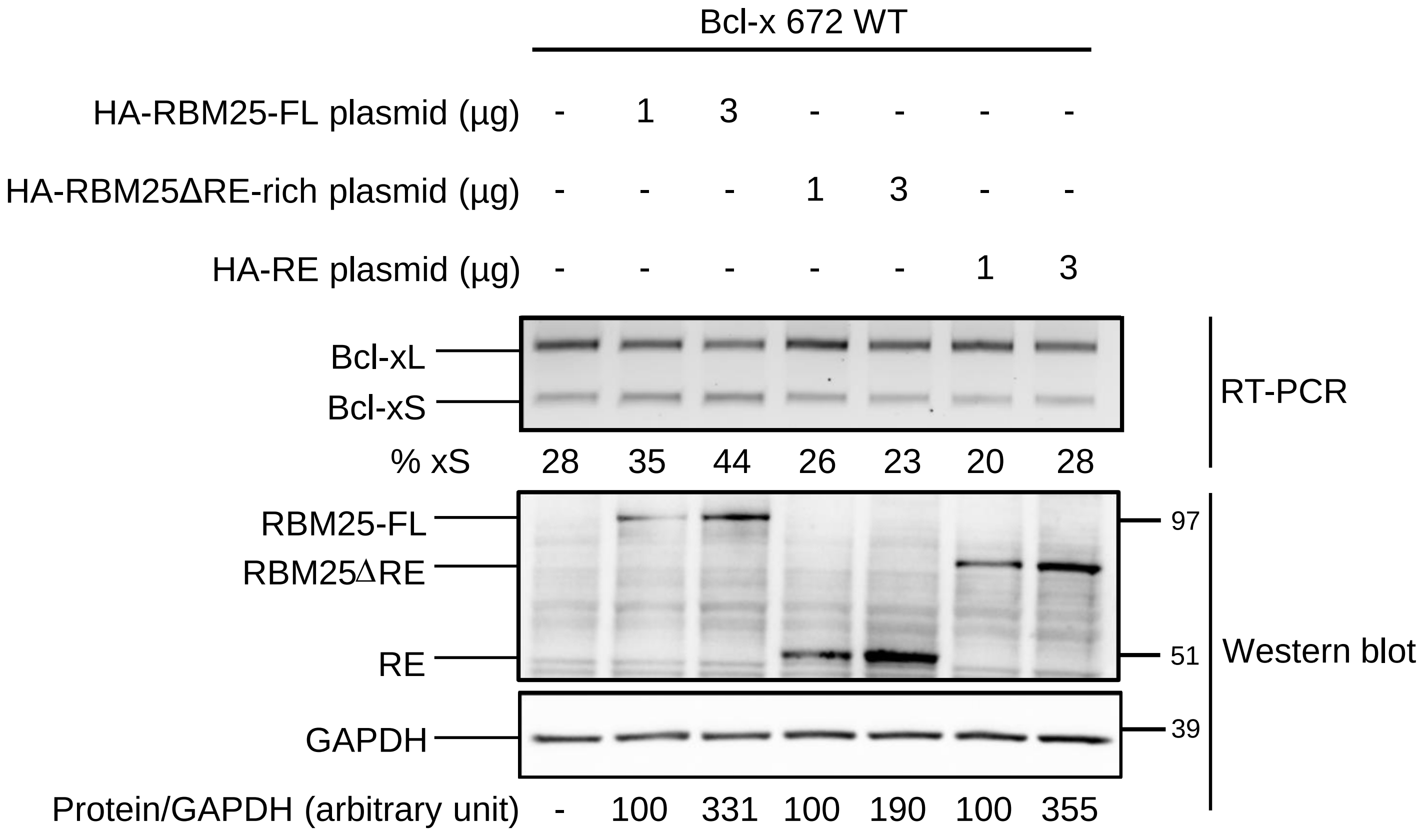

H1299 cells were transfected with the Bcl-x 672 WT minigene and a plasmid allowing expression of RBM25wt (HA-RBM25-FL), or a form of RBM25 deleted for its RE-rich motif (HA-RBM25-ΔRE-rich), or only the RE motif (HA-RE) to assess the effect of overexpressing one or the other of these various forms of RBM25 on alternative splicing from the Bcl-x 672 WT minigene. The result of the semi-quantitative RT-PCR experiment is shown in the upper panel and the western blot analysis of the level of expression of HA-RBM25-FL, HA-RBM25-ΔRE-rich or HA-RE, as compared to the loading control GAPDH, is shown in the two lower panels.

Supplementary Figure 7

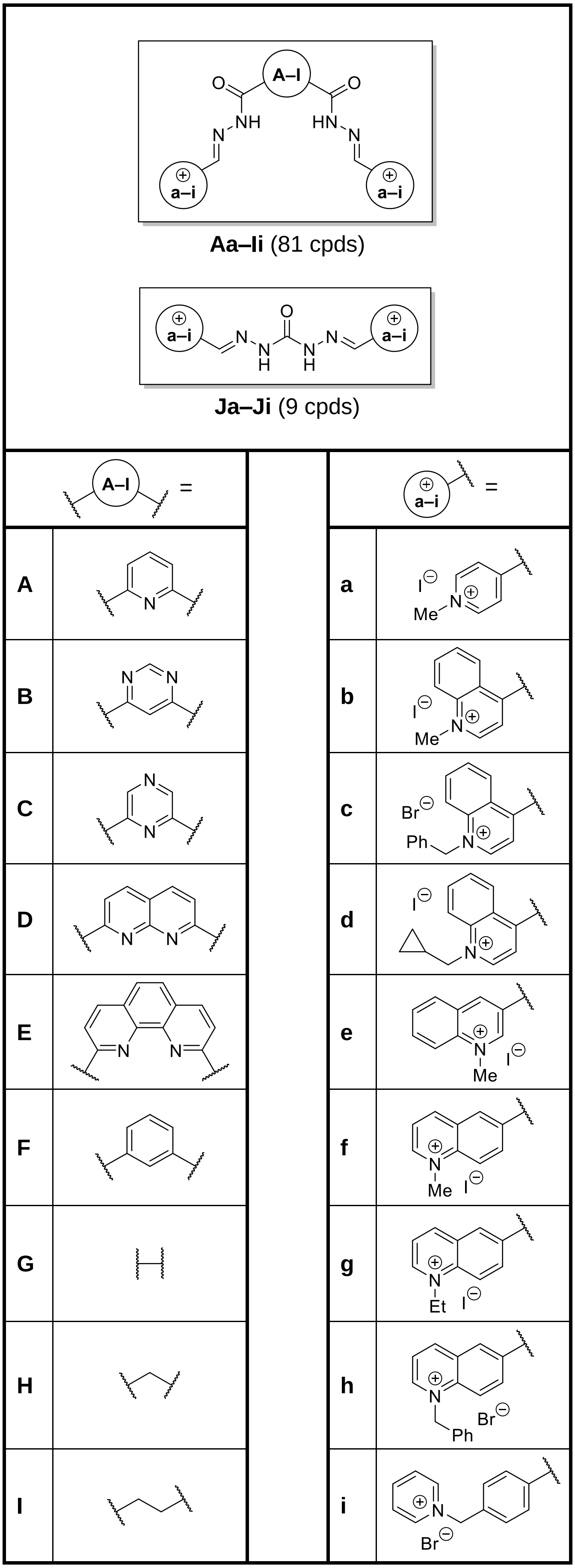

Combinatorial matrix of 90 cationic bis(acylhydrazones) screened as Bcl-x splicing modulators.

Supplementary Figure 8

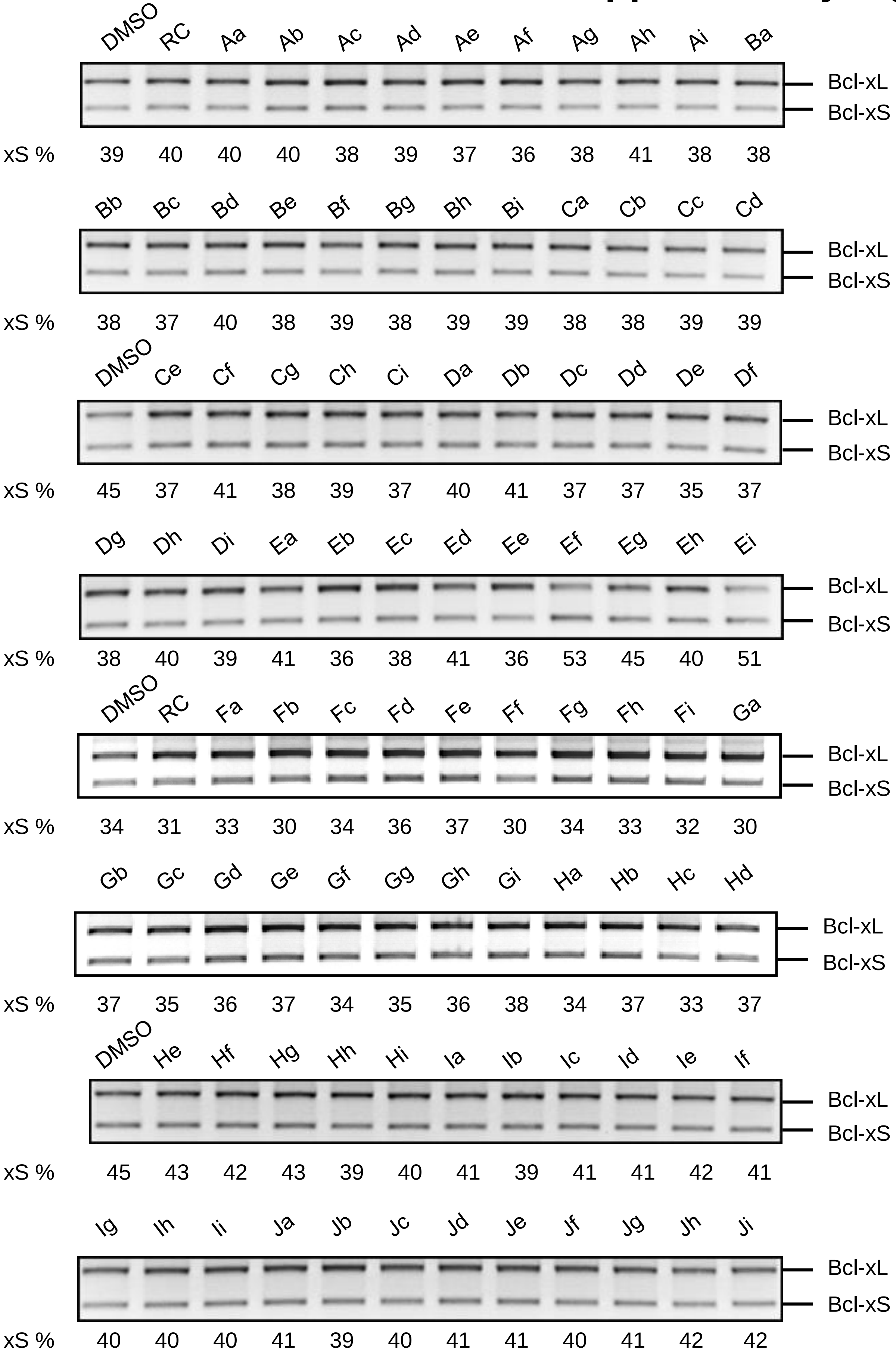

Screening of a combinatorial library of 90 "as-synthesized" cationic bis(acylhydrazones) structurally related to PhenDC3 and previously validated as putative G4 ligands (Reznichenko O et al *Chemistry* 2023) using a fixed concentration of ~10 µM and the splicing from the Bcl-x 672 WT minigene as a readout. The splicing from the Bcl-x 672 WT minigene was assessed using semi-quantitative RT-PCR experiments. DMSO and RC (Reaction Control, 1M AcOH in DMSO, a catalyst present in every sample) were used as negative controls.

Supplementary Figure 9

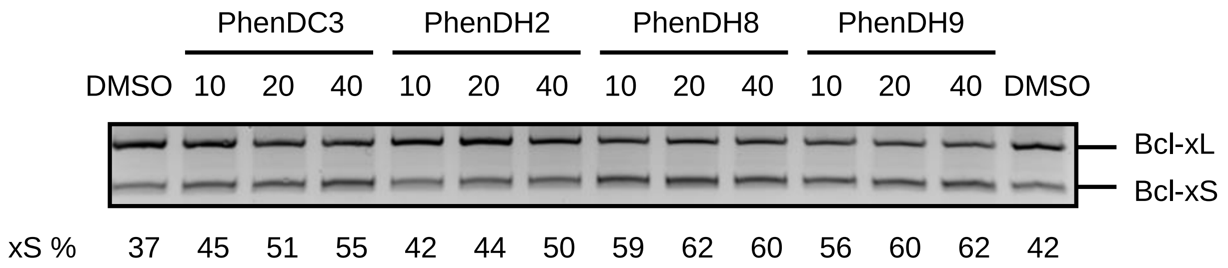

Effect of various concentrations of PhenD3, PhenDH2, PhenDH8 or PhenDH9 on the splicing from the Bcl-x 672 WT minigene as assessed using semi-quantitative RT-PCR experiments. DMSO (compounds vehicle) was used as a negative control.
